# IntensityCheck – The light measuring app for microscope performance checks and consistent fluorescence imaging

**DOI:** 10.1371/journal.pone.0214659

**Published:** 2019-03-28

**Authors:** Dirk Dormann

**Affiliations:** Microscopy facility, MRC London Institute of Medical Sciences, Faculty of Medicine, Imperial College London, London, United Kingdom; Arizona State University, UNITED STATES

## Abstract

Quantitative fluorescence imaging is an essential tool in biomedical research. It requires consistent and repeatable conditions such as constant sample illumination. Even on a confocal microscope this can usually only be achieved by using an external laser power meter. By combining low-cost wireless Arduino based light sensors with an easy to use Android smartphone app we provide microscope users with a simple but powerful tool to maintain sample illumination for quantitative imaging, for tracking the intensity, stability and alignment of the light sources and for comparing microscope performance.

## Introduction

Fluorescence microscopy and quantitative imaging are essential tools in current bio-medical research [1, 2]. While the operation of high-end fully automated microscopes like commercial confocal microscopes has become very easy it is still not so straightforward to illuminate the sample consistently with the same laser intensity over time as there are usually no built-in measurement devices that track laser output. Without resorting to the time consuming use of an expensive external laser power meter users can only hope that laser output and system alignment haven’t changed since their last imaging session if they wanted to compare fluorescence intensities with earlier recordings as it is often not possible or feasible to record all samples on the same day.

Regular quality checks of the imaging equipment by users or imaging facility staff are thus required to ensure the optimum and consistent performance of these sensitive instruments over long periods of time and measuring laser power and laser stability is at the core of that test regime as it probes the entire beam delivery path and its alignment [3–10].

To simplify the task of measuring laser power—or more generally fluorescence illumination light intensity–on the microscope and to provide users with an easy tool to achieve a constant sample illumination over extended periods of time the IntensityCheck light sensor and smartphone app were developed. The detection device only consists of a few low-cost electronic components (electronic components: ~£20; mechanical components: ~£20) and can be installed on any microscope, while the measurements are displayed on the user’s smartphone.

## Results

### Characterisation of the light sensors

The main consideration for developing IntensityCheck was ease of use, to have a light sensor mounted directly on the microscope to allow any user to measure illumination light intensity quickly without having to mount and align an external laser meter first. As we envisaged the sensors to be mounted permanently on the all confocal and high end instruments in a microscopy facility the required hardware components had to fit into a compact unit, had to be easy to assemble and cheap. We identified two suitable light sensors (TCS34725 and TSL2561) which were available mounted on circuit boards and a miniature Arduino compatible microcontroller (RFduino) for interfacing with the sensors and to provide wireless communication via Bluetooth [11, 12].

**Fig 1:**
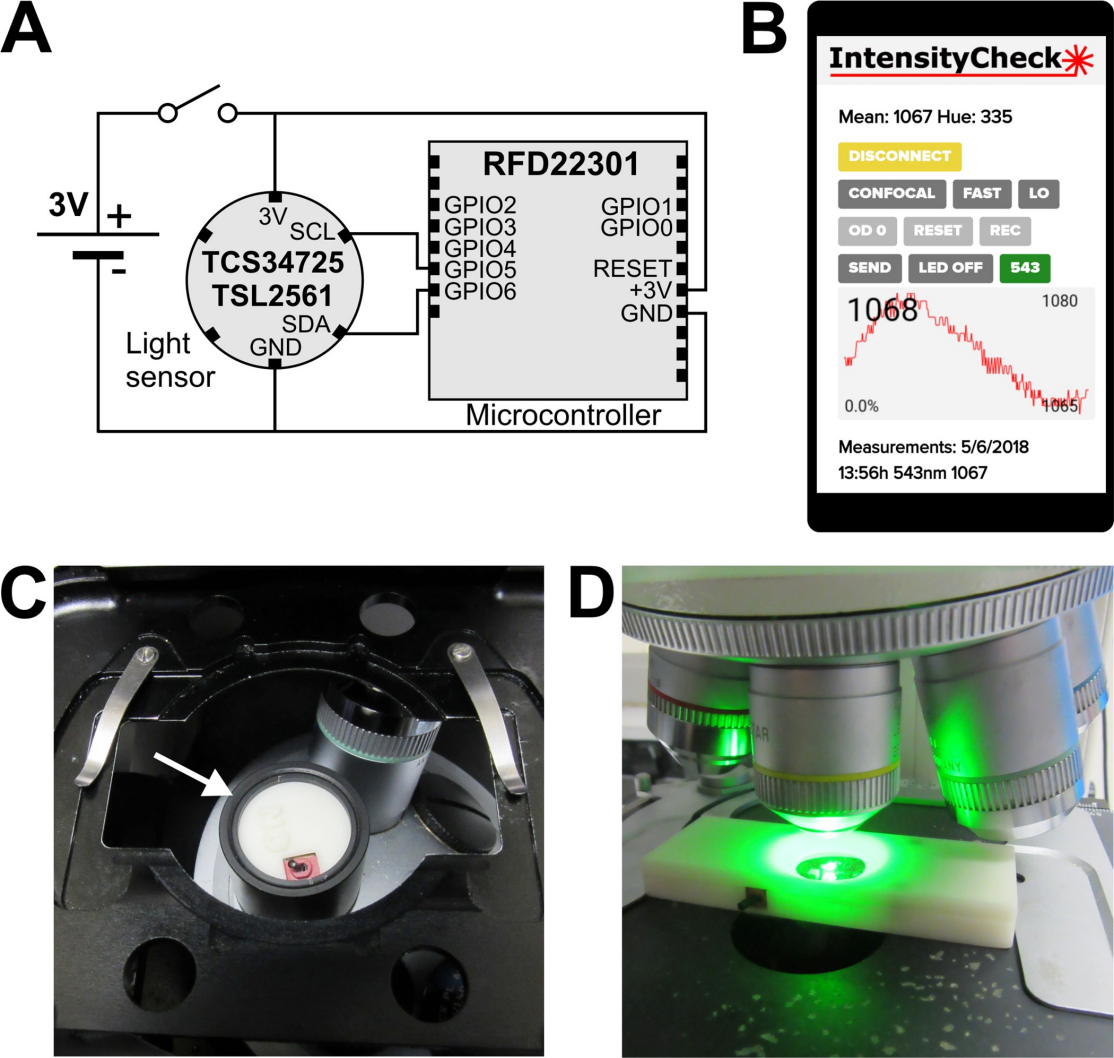
The IntensityCheck sensors and Android app. (A) Electrical wiring diagram of the TCS34725 or TSL2561 light sensor circuit boards to the RFduino microcontroller. (B) Smartphone screenshot of the IntensityCheck App. (C) The IntensityCheck sensor mounted as objective lens sized device on an inverted confocal microscope stand (arrow). (D) The slide shaped sensor on the stage of an upright fluorescence microscope. The pictures in (C) and (D) were taken by the author.

The electronic circuit is very basic (Fig 1A): sensor and microcontroller are connected via a two-wire I2C interface and powered by a 3V coin battery, with an additional on/off switch. Detailed instructions for the construction and use of the sensor units are available in S1 Text. The IntensityCheck app allows the user to display live information from the sensor on an Android smartphone, to adjust sensor integration time and gain and to record measurements (Fig 1B, S1 Video). The sensor electronics for the light detector have been packaged into two different units, either mounted like an objective lens directly on the objective turret of the microscope (Fig 1C, arrow) or as a slide-sized device (Fig 1D). The objective lens shaped device makes it particularly easy to switch between intensity measurements and imaging and was used to generate most of the data we discuss.

**Fig 2:**
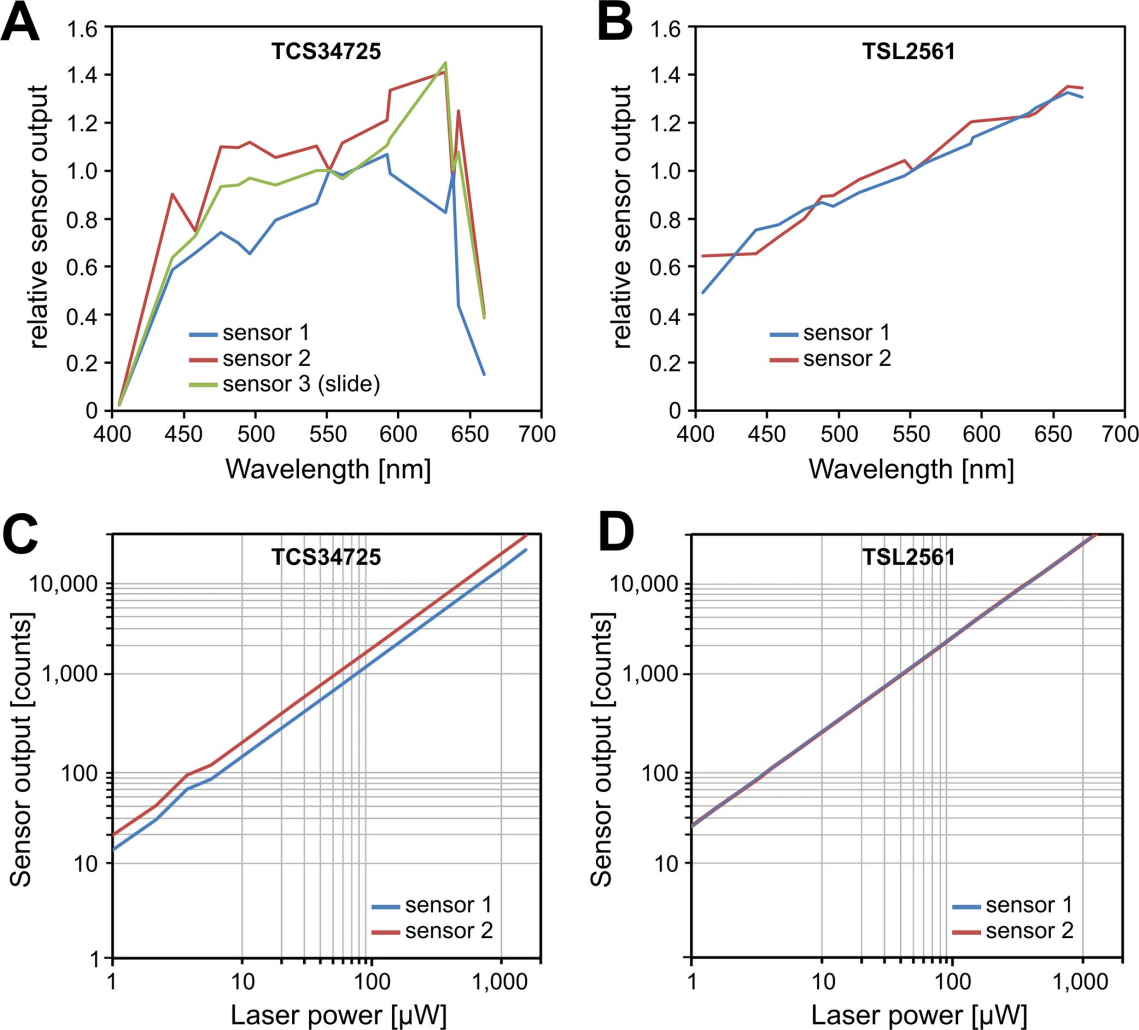
Comparing the spectral response and linearity of the different IntensityCheck light sensors. (A) Spectral response of three TCS34725 light sensors (two objective lens-shaped units mounted on objective turret, one slide sensor on the microscope stage) across the visible spectrum, with significant variation among the sensors. To compare the readings from the different sensors the output was calculated relative to the readings at 561nm. (B) Spectral response of two TSL2561 light sensors. (C) Measuring the linearity of the response of TCS34725 sensors 1 and 2 –same as in (A)—as a function of the actual laser power (0.2 to 1500 μW 488nm). (D) Linearity of the responses from the two TSL2561 sensors, which are actually overlapping in this plot.

To assess whether the sensors are suitable for the different light sources typically used in fluorescence microscopy (HBO, metal-halide, LED, lasers) we first characterised their spectral response in a wavelength range from 405nm to 670nm (Fig 2A and 2B). There was significant variation among the three TCS34725 sensors tested, but all exhibited a strongly reduced output towards the violet and red parts of the visible spectrum (Fig 2A), with the steep drop in the red probably due to the built-in infrared blocking filter. In comparison, the two TSL2561 sensors showed very little variation and a strong response in both the violet and the far red regions of the spectrum. Both sensor types showed a very linear response up to the mW range (~1.5mW) which would be relevant for most confocal applications, but the TCS34725 sensors displayed again noticeable variation (Fig 2C and 2D).

**Fig 3:**
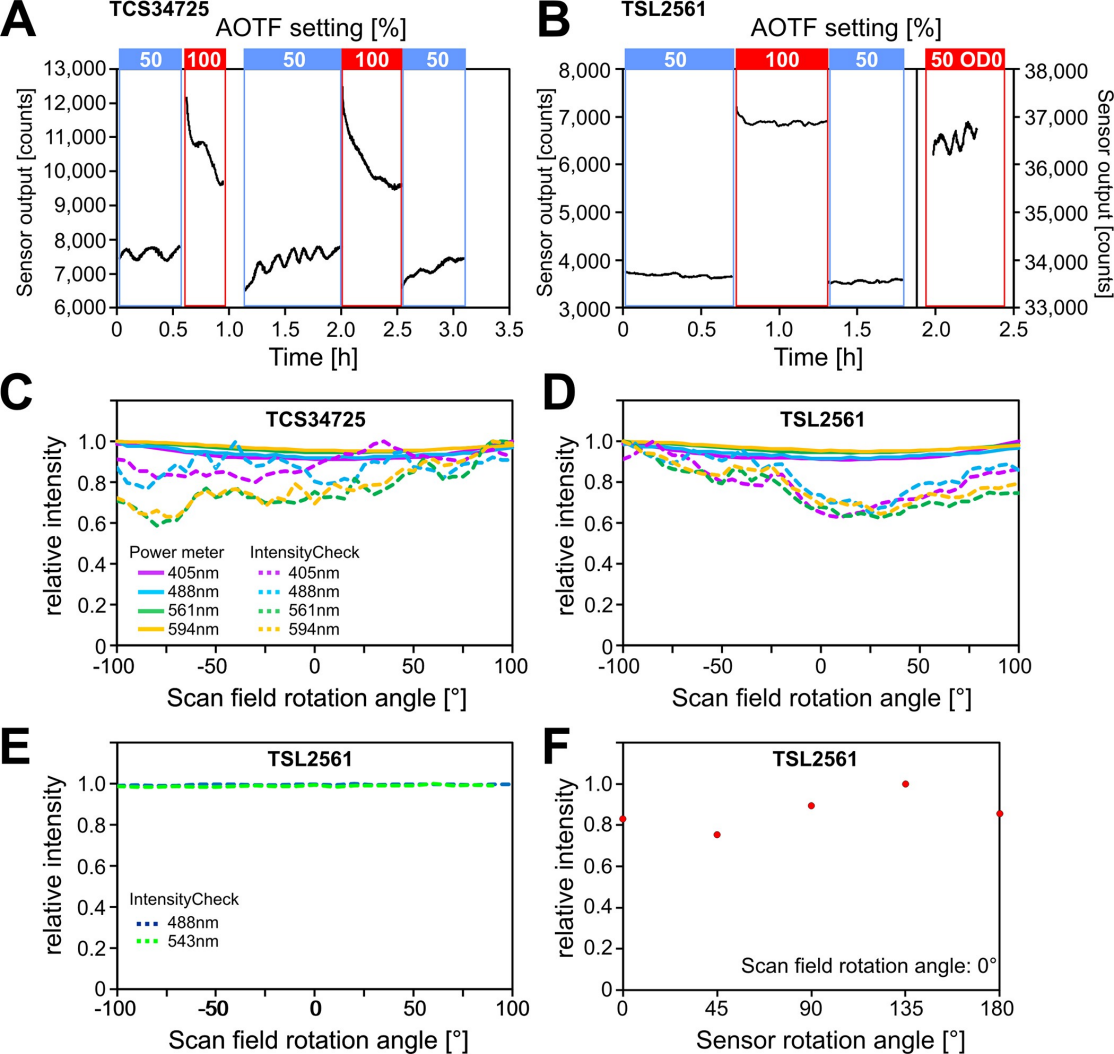
Factors affecting the light sensor output. (A) Timelapse recordings showing the effect of excessive laser light (561nm DPSS laser) on the output of a TCS34725 light sensor mounted behind an OD1 neutral density filter. At 50% AOTF the sensor detects small periodic laser fluctuations—presumably reflecting room temperature changes—but when power is raised to the maximum (100%) the signal drops rapidly. Upon reduction of laser power the sensor recovers again. (B) The TSL2561 (also with OD1 ND filter) didn’t show that response even when the AOTF was set to 100%. Even when the ND filter was removed, despite a 10fold increase in laser light (“50 OD0” setting) the sensor output didn’t drop; the 100% AOTF setting was not used as the sensor saturated just above 50%. Additionally the “FRAP-booster” option on this particular confocal model was employed to maximise the amount of laser light reaching the detector. (C,D) Effect of scan field rotation on sensor output on Leica confocal microscopes, shown for the two different sensors mounted on the objective turret (“IntensityCheck”), in comparison to power meter readings taken in the sample plane on the microscope stage. (E) On a different confocal instrument (Zeiss LMS510) rotating the scan area had no effect on the sensor output as the polarisation of the incident laser light is not changed by the rotation. (F) If the sensor itself is rotated on the same instrument at a given scan rotation angle–here 0°—the intensity output changes again depending on the angle.

Another issue which only seemed to affect the TCS34725 sensor was that excessive laser illumination led to a rapid decrease in sensor output (Fig 3A). Once laser power was reduced the sensor slowly recovered. As the sensor output was not even saturated at the higher laser power (100% AOTF), maybe the incident light didn’t just affect the light sensitive photodiodes but the sensor readout electronics which are all integrated together on the miniature silicon chip. Nevertheless this behaviour was not observed with the TSL2561 light sensor (Fig 3B).

We also noted that rotating the confocal scan field had a significant effect on the output of both sensors while the readings from the laser power meter barely changed (Fig 3C and 3D). On the Leica confocal microscopes used in this study the rotation of the scanned area is achieved by rotating an Abbe-Koenig prism which doesn’t preserve the polarisation of the laser beam as it rotates. This suggests that the response of the two light sensors might be depending on the orientation of the polarised laser light. This was tested using a Zeiss LSM510 confocal where the rotation of the scan area is achieved through the action of the two scanning mirrors thus maintaining the polarisation of the laser beam. And indeed, as the polarisation didn’t change during the rotation on this system (Fig 3E) the sensor response remained constant. If however the sensor was rotated the intensity changed (Fig 3F).

**Fig 4:**
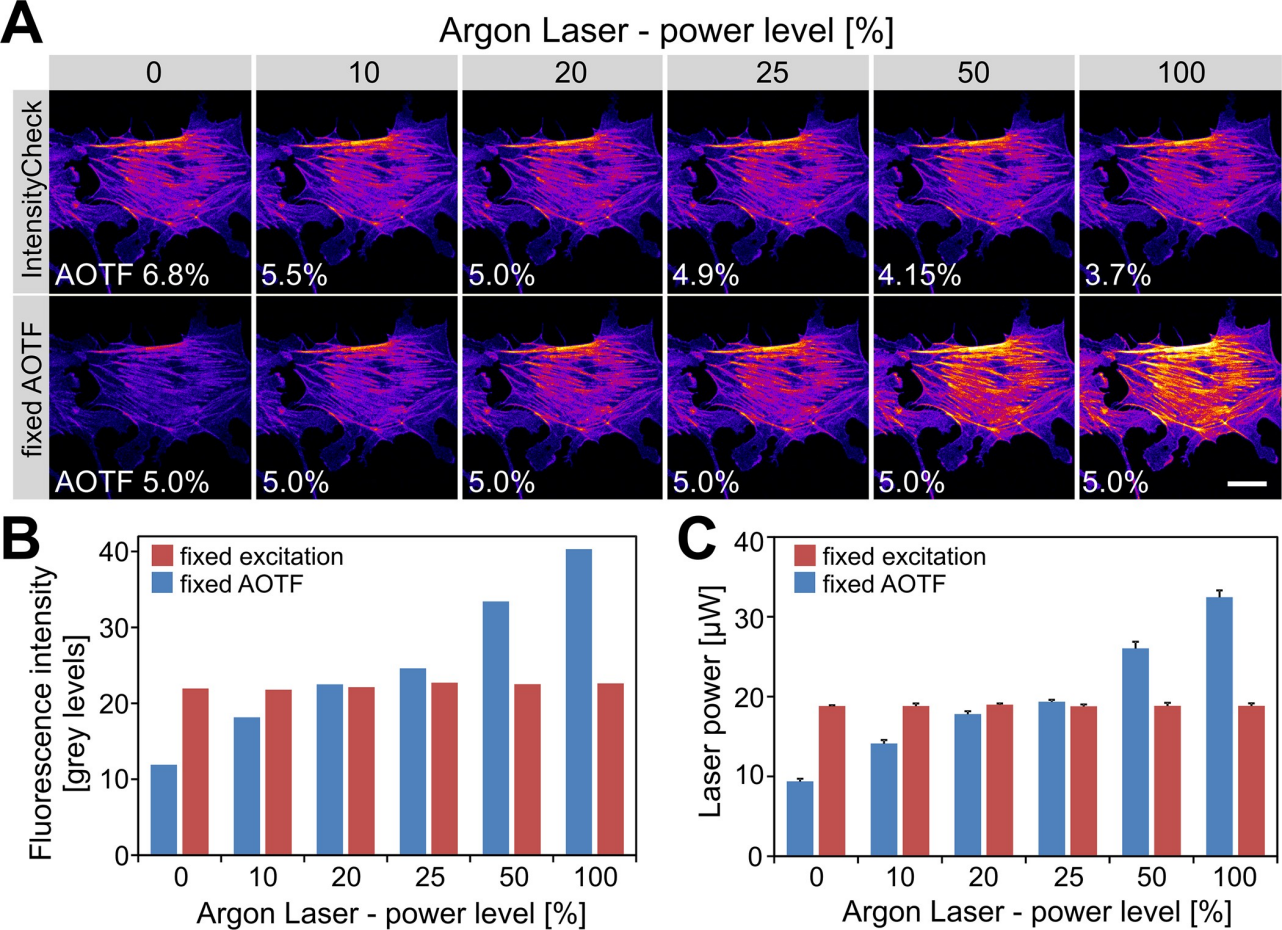
Using IntensityCheck to maintain constant sample illumination. (A) The upper row shows the constant sample fluorescence achieved with IntensityCheck despite ramping the Argon laser output “power levels” (488nm) from 0% to 100%. The actual AOTF settings are also displayed. The lower row for comparison with a fixed 5% AOTF setting shows an increase in fluorescence due to the rising laser output. Pseudo-colour representation of Alexa488-phalloidin labelled bovine endothelial cells. Scale bar: 20 μm. (B) Quantification of the average image intensities from (A). (C) Measuring the actual laser power in the sample plane on the microscope stage in a separate experiment. The target for IntensityCheck here was to maintain 18.8 μW (488nm) while increasing the laser output (0 to 100% power level). Achieved was 18.85 ± 0.25 μW (n = 30; using theTCS34725 colour sensor). Shown are mean laser power and standard deviations of five attempts per laser power level to reach the target value with IntensityCheck.

Overall the TSL2561 sensor provided the best performance with a strong response over the visible spectrum while not suffering from any detrimental effects when exposed to strong illumination and was therefore used for most experiments. The TCS34725 sensor was more limited in its spectral response, but has the ability to sense colours through red, green and blue sensitive photodiodes. The IntensityCheck app takes advantage of that to detect the wavelength of the fluorescence excitation light (S1 Video). The TCS34725 sensor board also contains a white LED which we used to characterise the confocal detectors (see below). To cope with higher laser power appropriate neutral density filters should be mounted in front of the sensors (Fig 4 in S1 Text). The effect of polarisation on the intensity readings can be mitigated on the Leica confocal microscopes by first adjusting the scanfield rotation to maximise the sensor output.

### Using IntensityCheck to maintain constant sample illumination

Having established that the sensors are suitable for measuring laser intensity we examined how IntensityCheck can be used to obtain constant sample illumination on a confocal microscope for quantitative imaging.

We first recorded images of a standard biological sample (Alexa488-phalloidin labelled cells, FluoCells) at 5% AOTF (acousto-optical tunable filter; 488nm laser line) and 20% laser power (corresponding to the Argon laser tube current)–a typical setting on our Leica confocal microscopes when using sensitive hybrid detectors. After changing from the 20× imaging objective to the IntensityCheck sensor mounted on the objective turret (Fig 1C) the corresponding light intensity was measured. This represented the target value we aimed to achieve for the subsequent recordings. Variations in the actual Argon laser output were created by ramping up the laser power levels between 0% and 100%. At each power level the AOTF was first adjusted with IntensityCheck to obtain the target value, directly followed by re-imaging the same sample; for comparison images were recorded with a fixed 5% AOTF setting. The fluorescence intensities of the sample cell images remained indeed constant when adjusted with IntensityCheck (Fig 4A and 4B). Measuring actual laser intensities in a separate experiment (Fig 4C) gave similar results, confirming the feasibility of this approach to keep sample illumination constant.

### Reducing the sample illumination variability over longer periods of time

Although we changed the Argon laser output above on purpose—as a proof of concept -, the temporal variation of laser power in confocal instruments is well documented and often not just due to the lasers themselves but due to the alignment and calibration of many components in the excitation light path as well as environmental factors like temperature [3, 4, 8, 9].

**Fig 5:**
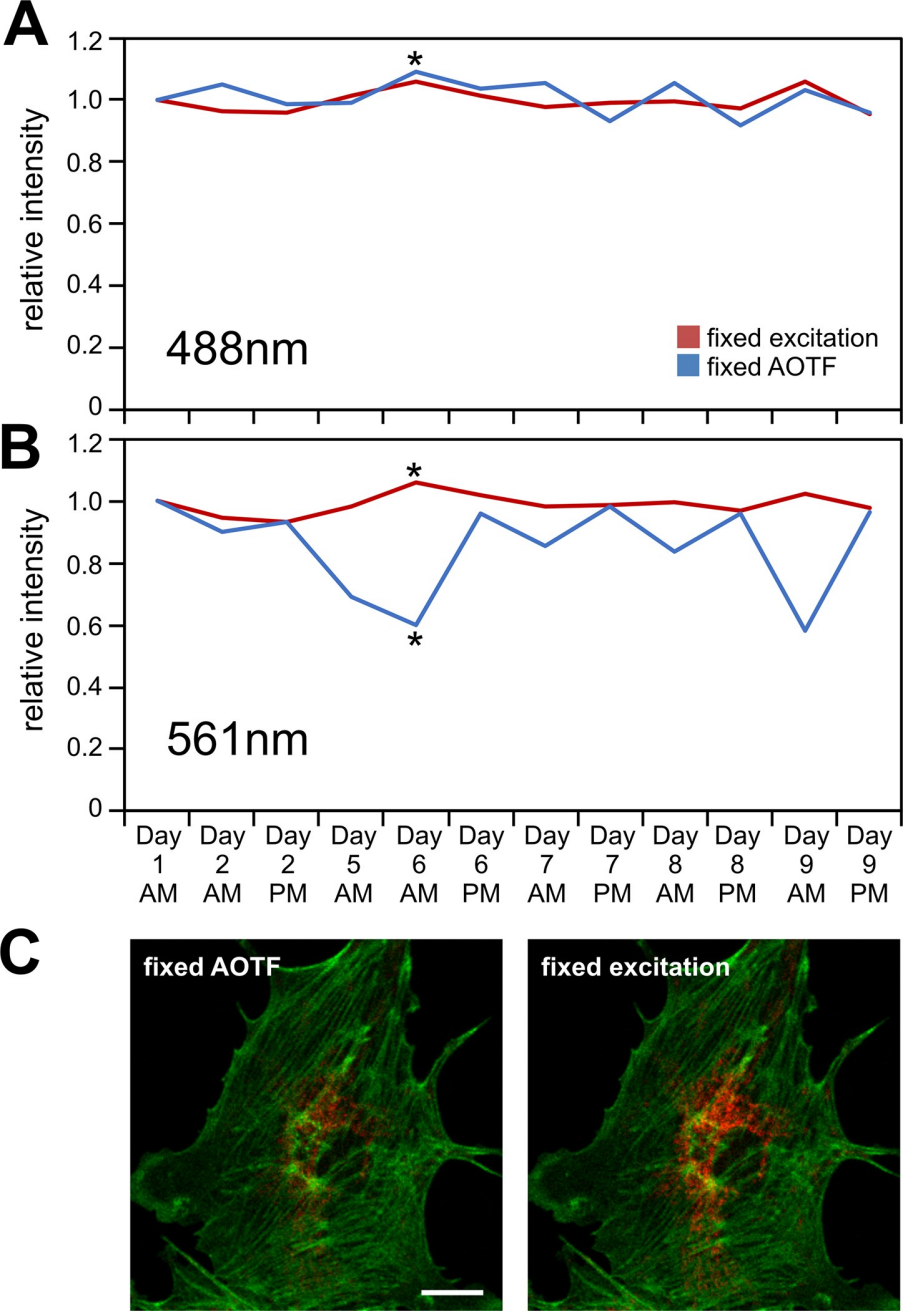
Imaging and correcting the day-to-day confocal laser variability. (A,B) The same FluoCells cells were imaged over a number of days with either fixed laser excitation (using IntensityCheck) or using the fixed 5% AOTF setting for the 488nm Argon laser and 561nm laser. Plotted are the mean fluorescence image intensities relative to the intensities on day 1; AM/PM denote morning and afternoon recordings, typically taken 4–6 hours apart. The asterisks mark the timepoint on day 6/AM as panel (C) shows the corresponding fluorescence images with the red mitochondrial label barely visible in the uncorrected image (“fixed AOTF”). Scale bar: 10μm.

To demonstrate that IntensityCheck is a useful tool to counter-act that variability we carried out short and long term experiments. The confocal day-to-day variability was tested by imaging fluorescent Alexa488-phalloidin and MitoTracker Red labelled cells. The cells were imaged on day 1 (settings: 5% AOTF/488nm, Argon laser power level: 20%; 5% AOTF/561nm), immediately followed by measuring the corresponding target intensities with IntensityCheck. At all subsequent time points we recorded the same cells again with two different settings: either using the same AOTF settings as on day 1 (5%, “fixed AOTF”, Fig 5) or the IntensityCheck corrected settings based on the original target intensities (“fixed excitation”, Fig 5).

Although there was actually very little variation from the Argon laser–here quantified as the relative mean intensity of the fluorescence images–the IntensityCheck based images showed even less variability (Fig 5A). In contrast the uncorrected 561nm excitation changed significantly (Fig 5B and 5C) presumably reflecting corresponding changes in laser output.

A long enough warm up time of the lasers and the instrument (> 2h) reduced some of the variation of the 561nm laser: on days 6 and 9 both the instrument and the lasers were switched on shortly before imaging in the morning (AM, Fig 5B and 5C) resulting in very dim MitoTracker images while the 2^nd^ set of images recorded 4 to 6 hours later were much brighter (PM, Fig 5B and 5C). On days 2, 7 and 8 the instrument had been running since the previous day and only the lasers were turned on in the morning; the difference between the AM and PM values was much reduced on those days indicating that status of the instrument itself can make a significant contribution.

However even in a busy multi-user microscopy facility not all confocal lasers are switched on all the time, instruments might be turned off inadvertently and confocal access might be very limited not allowing enough time for particular lasers to warm-up sufficiently. Although users often re-load previously used acquisition settings in an attempt to re-create previous imaging conditions, the data presented suggest that there can be considerable daily variation in sample illumination which can be mitigated by measuring laser power or using a tool like IntensityCheck.

**Fig 6:**
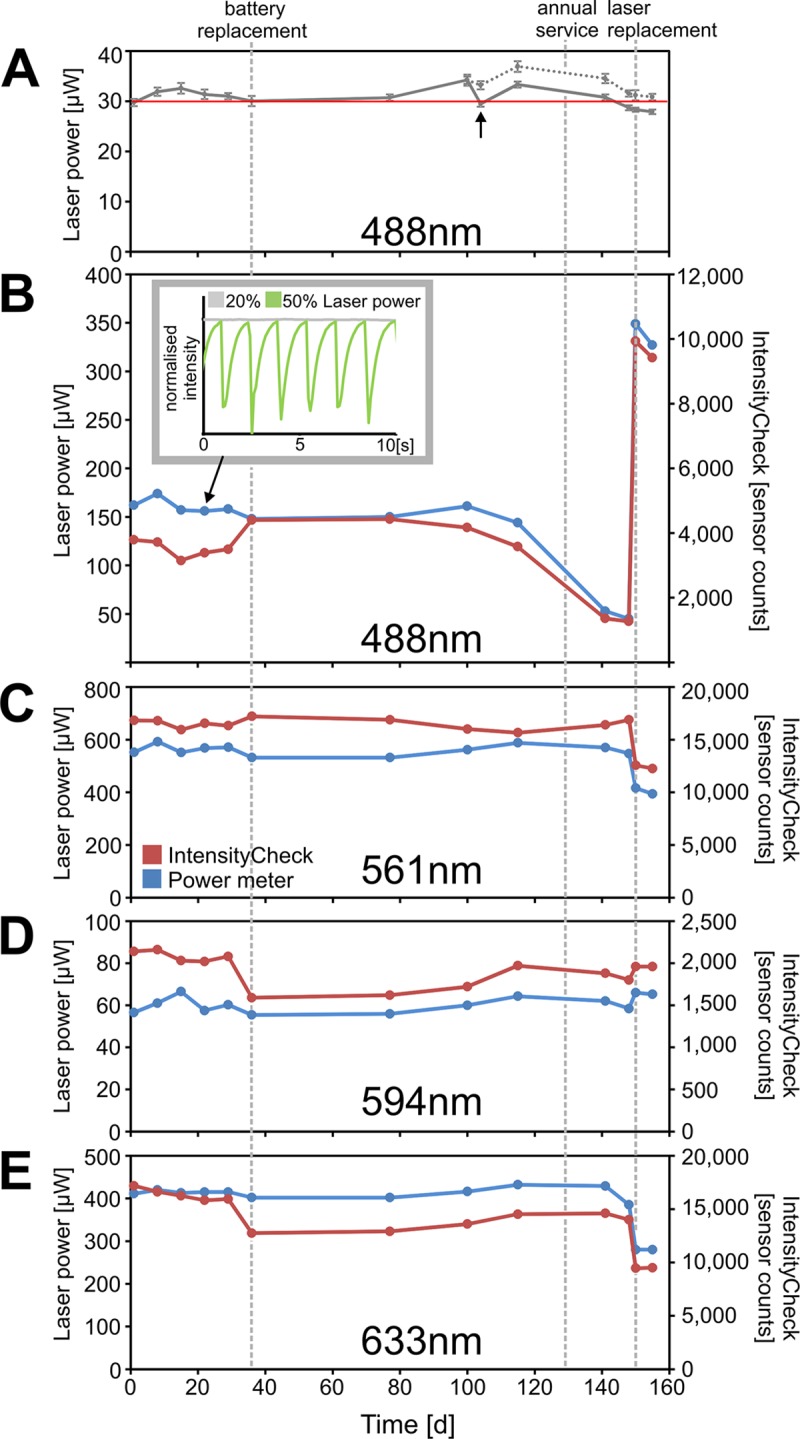
Long-term laser power measurements using IntensityCheck. (A) IntensityCheck was used to maintain an arbitrary sample illumination intensity of 30 μW (488 nm) despite the large variations in laser output shown in (B). Recalibration was required after battery replacement (changing IntensityCheck target value from 680 to 910), and again after the actual laser power had changed by more than 10% (arrow). The dotted line shows the laser power if the second recalibration had not been carried out. (B) At the same time points IntensityCheck readings were compared to the actual laser meter readings measured in the sample plane of a confocal microscope (488nm line of the Argon laser, laser power level: 20%; AOTF: 25%; TSL2561 light sensor). IntensityCheck followed the measured laser output well, except for a sudden change when the device was removed from the objective turret for battery replacement, probably slightly displacing or tilting the sensor in the process compared to the previous time points. The inset shows IntensityCheck recordings taken at the indicated timepoint (arrow) with abnormal laser oscillations at high tube current indicative of an aging gas laser that eventually failed requiring replacement. (C-E) Corresponding measurements of the other lasers on the system: 561nm DPSS, 594nm HeNe laser and 633nm HeNe laser. For each laserline the scan field rotation was adjusted first for maximum detector output.

In a long-term study we established that the IntensityCheck sensor can maintain a set illumination intensity over five months (30μW at 488nm; Fig 6A). Over the same time course we compared laser meter readings with the IntensityCheck output (Fig 6B–6E). Despite the huge variations in the Argon laser output (Fig 6B), the sample illumination intensity was kept at 30μW (30.7±1.9 μW; Fig 6A) but re-calibration was required on two occasions: after battery replacement and—as a precaution—after the intensity had changed by more than 10% compared to a previous reading (see arrow in Fig 6A).

Although the scan field rotation was always adjusted prior to taking measurements the significant change in all the IntensityCheck laser measurements following the replacement of the sensor battery (Fig 6B–6E) indicated that others factors—maybe a small tilt of the whole sensor assembly or off-centre mounting of the sensor–might be responsible. This could have happened as in the original sensor design used for this experiment the entire sensor unit had to be first removed from the metal casing to access the battery and then re-inserted back into the metal holder (Fig 4 in S1 Text). To avoid this potential issue we have subsequently redesigned the sensor assembly to give direct access to the battery and to ensure the TSL2561 sensor is centred as much as possible (S1 Fig, S1 Text). Changing the battery has no longer an effect on the sensor output (S1 Fig). With these safeguards users should be able to achieve consistent imaging conditions over many months.

Apart from the battery replacement issues discussed above the IntensityCheck results generally followed the laser meter readings quite well, indicating that the IntensityCheck sensor can indeed replace an external power meter (Fig 6B–6E). The deterioration of the Argon laser performance with increasing tube current was first noticed as oscillations on the live display of the IntensityCheck App (Fig 6B, inset). The Argon laser output dropped from day 100 and eventually had to be replaced (Fig 6B). The output from the other built-in lasers changed as well, presumably due to laser re-alignment by the service engineer as all visible laser lines are merged (Fig 6C–6E).

### Further applications of IntensityCheck

Changes in laser output happen not only over longer time scales as shown above but small intensity fluctuations can occur on a much shorter scale [4, 8]. While this can be picked with the confocal transmitted light detector, IntensityCheck can detect the same short-term laser power fluctuations due to sensor readout speeds between 100 ms and 700 ms (S2 Fig).

The illumination light intensity can of course be measured on other fluorescence instruments as well. We followed laser power over time on a custom-built TIRF (total internal reflection fluorescence) microscope (S3A Fig) and were able to maintain set laser intensities (S3B Fig). This could potentially be useful to ensure reproducible imaging conditions whether for live cell TIRF experiments or when performing single molecule localisation experiments where particular laser intensities in the 2–10 kW/cm^2^ range are required to induce the blinking of the photo-switchable dye molecules [13]. Furthermore IntensityCheck can be used to assess the output and stability of conventional fluorescence light sources (short arc mercury bulbs or LED) on widefield microscopes and for alignment purposes (S4 Fig).

### Characterisation of the confocal detectors

As the circuit board of the TCS34725 colour sensor also carries a small LED we wondered whether that would be a suitable light source to compare the performance of the confocal detectors. Due to the positioning of the LED roughly in the back focal plane—when using our objective shaped design—an objective lens isn’t even required in contrast to previously proposed methods which relied on a stage mounted light source [14]. The white light emitted by the LED showed a very broad spectrum, was very stable and uniform when imaged with the confocal scanner (laser light off) (S5 Fig).

For the actual experiments we started with low scan frequency and open pinhole to collect a large amount of the light emitted by the LED (see Materials and Methods). The gain was adjusted for each confocal detector to achieve a mean image intensity of about 180 (out of 256 grey levels to avoid saturation). Given the stable light input we then varied those parameters that would typically affect the confocal image contrast or the signal-to-noise ratio (SNR): scan frequency, pinhole diameter and frame averaging. Using the same IntensityCheck device images were recorded for all detectors on the four Leica confocal microscopes available in the facility and the SNR was calculated by dividing the mean image intensity by the standard deviation.

**Fig 7:**
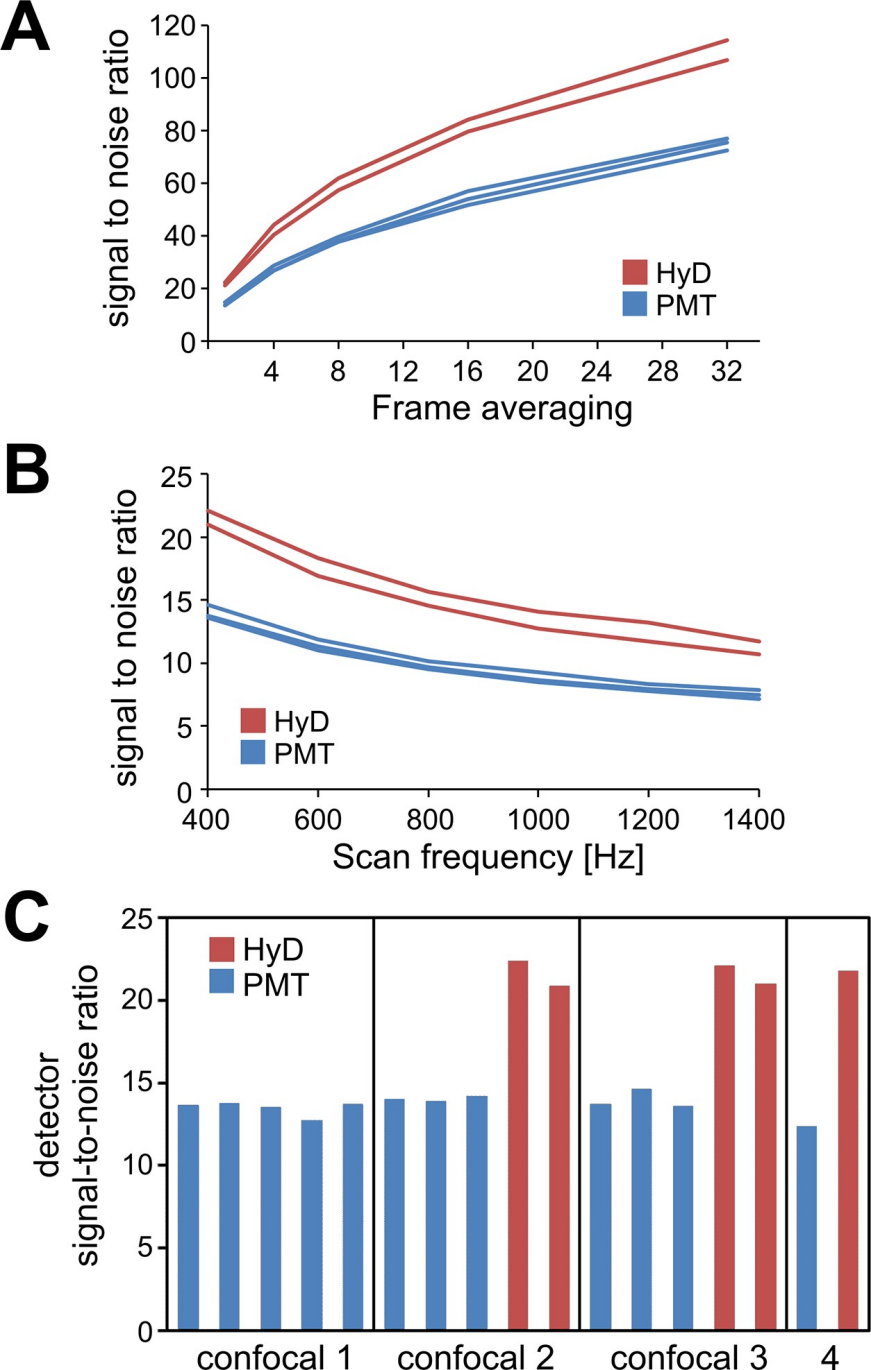
Comparing the performance of different confocal light detectors across multiple instruments. (A) Effect of frame averaging on the signal-to-noise ratio of the five detectors on a Leica confocal microscope. (B) The decrease in pixel dwell time due to the increase in scan speed causes the reduction of the signal-to-noise ratio—same detectors as in (A). (C) The signal-to-noise ratio of images acquired with the stable output from the LED mounted on the colour sensor circuit board is useful to compare and characterise different confocal detector types (photomultiplier tubes, PMT, versus hybrid detectors, HyD) across multiple Leica confocal microscopes. The HyD detectors provide a significant improvement over the photomultiplier tubes.

Fig 7A shows the effect of frame averaging on the five detectors of a confocal microscope. The SNR improved proportional to the square root of the number of frames averaged of this confocal microscope [15]. Significant differences were observed between detectors types: the HyD hybrid detectors with their higher quantum efficiency and lower noise floor performed better than the conventional photomultiplier tubes (PMT). When the scan frequency was increased the SNR dropped due to the shorter pixel dwell time but the difference between detector types remained (Fig 7B). With this approach we could actually compare detectors across systems, and indeed the detectors performed very similarly on different microscopes (Fig 7C). Comparing the detectors of confocal microscopes from different manufacturers remained however challenging due to the difficulties of recreating equivalent imaging conditions across platforms (due to pinhole geometries, dichroic mirrors, optical filters versus spectral detectors, pixel dwell time etc). Nevertheless a stable light source like this mounted directly on the objective turret should help towards that aim and could be useful in assessing detector performance over time.

## Discussion

The IntensityCheck tool presented here should benefit many microscopists and imaging facility staff alike for both routine system checks and demanding fluorescence imaging applications. By utilising a smartphone as readout device every microscope user has the ability to measure and adjust the illumination light intensity consistently in a very simple manner, significantly reducing the variability of the instrumentation. Due to the low cost of the individual devices we would envisage the units to be mounted on every high-end system in a facility where the illumination intensity need to be monitored. Even without access to a calibrated power meter the IntensityCheck device is useful for measuring relative intensity changes over time, for alignment purposes or where a remote wireless readout might be desirable. Although currently not implemented the Android app could be extended further by uploading intensity measurements directly to a webserver, for example to track laser performance over time which would further simplify the workflow for facility staff.

Apart from issues like the apparent sensitivity of the sensor output to the direction of the polarised laser light, which can be mitigated as discussed above, a major drawback is the small size of the relevant light sensitive areas on the sensors (TCS34725: ~0.144mm^2^; TSL2561 ~0.8mm^2^). While in confocal imaging the back focal plane of the objective is filled or often even overfilled by the expanded laser beam to achieve a diffraction limited excitation spot in the focal plane, in widefield or TIRF imaging the laser beam is focused on the back focal plane. Consequently the objective turret mounted IntensityCheck sensor would detect various fractions of the total illumination beam. To capture all the light at the back focal plane additional focussing optics would be required; however we could not find a solution to integrate this with the current electronics within the space constraints of an objective lens sized sensor. If this could be overcome it would be feasible to calibrate the sensors in a wavelength dependent manner against a laser power meter to provide absolute power measurements.

Another limitation of our approach to maintain constant sample illumination is of course that the user has to adjust the light intensity manually, therefore any short term fluctuations in the output of the light source (sub-second to seconds or even minutes) cannot be mitigated in this way. Ideally there should be a sensor that picks up a small amount of the excitation light via a beam splitter somewhere close to the objective lenses, maybe similar to where modern hardware autofocus units are placed, just below the objective turret. The sensor would continuously monitor light intensity and feedback electronically to the AOTF, a directly modulated laser or the LED drivers to compensate any deviation from the set intensity. While this would be very desirable and should be incorporated by instrument manufacturers, this is currently not the case so the user has to take action and measure the illumination light intensity where appropriate.

Many studies and our own observations presented here have shown that the illumination light intensity can change over time, over the range of hours—when the instrument is first switched on—to changes over weeks and months [3, 4, 8]. As our day-to-day variability assessment exemplified (Fig 5), albeit it with a bright standard sample, this should not be ignored and it does make quantitative imaging challenging.

For quantitative comparisons all samples should of course be imaged with the appropriate controls during the same imaging session on an instrument where the hardware and the light sources have had sufficient time to warm-up and stabilise. If however this cannot be achieved, for example due to the large number of samples to be imaged, due to the availability of cell lines or animals or when replicate experiments need to be carried out at a later time, there could be a considerable uncertainty over the actual output from the different lasers. In the worst case differences in the sample fluorescence could be due to the altered illumination intensity rather than reflecting the biology (Fig 5). Any modest changes in protein expression levels for example (maybe 10–20%; quantified through fluorescent proteins or antibody staining) could easily be missed or misinterpreted if the illumination intensities vary on a similar scale. Similar issues might occur when two different light sources are required, as for ratiometric or FRET (fluorescence resonance energy transfer) studies; any relative intensity change in one or both of the channels could impact on the results and the conclusions drawn.

When conducting live cell imaging, in particular high resolution time lapse studies, those experiments would typically be carried out sequentially over a number days or weeks. Variations in the excitation light would cause changes in the apparent fluorescent signal, which could make the observation of the structure in question or any quantitative comparisons difficult. But live cells are also very sensitive to photo-toxic effects induced by the strong excitation light, thus an unnoticed rise in light intensity could potentially damage the cells and lead to un-physiological responses.

These examples illustrate the importance of monitoring and maintaining constant illumination throughout the course of an experiment or study, using either a power meter or a simple tool like IntensityCheck.

And finally we would propose that the open-source hard- and software framework provided here for wireless communication between electronic devices might be useful for the wider research community. It can easily be adapted and expanded to interface with different sensors or to control completely different instruments, all by tapping on the screen of a smartphone.

## Materials and methods

### Sensor characterisation and laser power measurements

The IntensityCheck light detectors were assembled and used as described in S1 Text. The experiments were carried out on the four Leica confocal microscopes available in the microscopy facility (Leica SP5, SP5II, SP8 STEDX, SP8 DLS) and a number of customised widefield microscopes (Leica DMRB, Leica DMI6000, Olympus IX70/71, Zeiss Axio Observer Z1, Zeiss Axiovert 200, Nikon eclipse E400), with a variety of fluorescence light sources (100W HBO mercury short arc lamps, Leica EL6000 metal halide lamps, Lumencor Spectra solid state light source, Cairn OptoTIRF with Cairn MultiLine Laserbank 405nm/462nm/635nm lasers).

Laser safety: Observe all local instrument specific and departmental laser safety rules to prevent any accidental release of laser light when using, handling or mounting the detector devices. The devices have only been developed for typical confocal imaging conditions and might not withstand high power laser beams.

For all confocal laser power measurements the ‘bleach point’ mode was used with a stationary beam parked in the centre of the field of view to avoid the oscillations caused by the blanking of the laser beam typically encountered during conventional 2D scanning [4, 5].

The spectral response of the sensors was determined on a Leica SP8 STED X confocal system using a wide range of commonly used laser lines ranging from 405nm to 670nm. For each wavelength the laser power was first adjusted to 100 μW with an external stage-mounted power meter (Fieldmaster power meter, Coherent Inc., with LM-2 VIS sensor), followed by measuring the output from the different sensors. To compare the linearity of the IntensityCheck sensors with the laser power meter we measured intensities of a 488 nm Argon line between 0.2 μW and 1.5 mW. The effect of scan field rotation on the sensor output was assessed by measuring laser output for a fixed AOTF setting while varying field rotation from -100° to +100° in 5 degree steps on the Leica confocal microscopes. Due to the observed effect of scan field rotation on the IntensityCheck sensor output (Fig 3C and 3D) we optimised the field rotation for each laser line to maximise the readout before taking the actual measurements as this provided the most consistent results when compared with the power meter. For the long term confocal laser power measurements a neutral density filter (OD 1.0) was mounted in front of the TSL2561 sensor, for each laser the AOTF was always adjusted to 25%, with the Argon laser power level set to 20%.

Short term laser noise was measured with the IntensityCheck sensors in time lapse mode and with the confocal transmitted light detectors as described [3, 4].

The high laser power of the TIRF setup was measured on the microscope stage using a Thorlabs S175C microscope slide thermal power sensor connected to a PM100D digital optical power meter.

### Comparing confocal detectors

The spectral properties of the LED mounted on the colour sensor board used for comparing the signal-to-noise ratio of the confocal detectors were measured with a lambda scan using the spectral detection of the Leica confocal microscopes. The stability of the LED output was measured by the light sensor itself due to the back reflection of some of the LED emission light from the neutral density filter mounted in front of the detector board (see Fig 4 in S1 Text). Although switching on the LED only led to an additional current of 2–3 mA a constant light output could not be maintained with the CR 2032 3V battery used (S5B Fig, inset), presumably as those small coin batteries are not designed to support high load currents for extended periods of time. This was resolved by building a device where the sensor, mounted in the lens tube on the objective turret, was connected via a long ribbon cable to a RFduino unit (RFD22102) mounted directly on the USB programming module (RFD22121) thus relying on the stabilised power supply provided by the USB connection to the computer. 20min after turning on the LED the output was stable enough to capture the images, at maximum zoom settings of the confocal scanner (48× or 64×) to achieve a homogeneous scan field (64 × 64 pixels). The gain for each detector was adjusted to achieve a mean pixel intensity of 180 (256 grey level range; offset 0V) to avoid saturation, pinhole diameter was set to 4 airy units for a 10x/0.4NA objective lens, detection wavelength range 450–650 nm. Image series were recorded for each detector and the relevant scan parameters were systematically changed: scan speed (400/600/800/1000/1200/1400Hz), frame averaging (1/4/8/16/32× averaging), pinhole diameter (reducing the amount of light reaching the detector) (4/3/2/1.5/1/0.75/0.5/0.38 AU). The signal-to-noise ratio was calculated by dividing the mean image intensity by the standard deviation.

### Maintaining constant illumination intensity

The basic procedure to maintain a consistent illumination intensity over time was the following:

1. Image the sample with the required objective lens.
2. Rotate the microscope objective turret to bring the IntensityCheck detector into the light path.
3. Measure the corresponding sensor readout with the IntensityCheck App. This is the target intensity that needs to be achieved for that particular objective lens and wavelength for future imaging sessions. Use the ‘bleach point’ mode described above for the IntensityCheck measurements to avoid fluctuating readouts.
4. At the next imaging session rotate the IntensityCheck detector into the light path first. Please note that laser output can change significantly when the lasers have just been switched on, they might need 90–120 min warm-up time to stabilise sufficiently [3, 4].
5. Adjust the laser intensity until the target value is reached on the IntensityCheck App display (Use ‘bleach point’ mode!).
6. Change to the objective lens and image the sample.

Several precautions should be taken:

1. The objective lenses should always be clean, otherwise the procedure would be invalidated as the lens is bypassed during the intensity measurements.
2. Due to the sensitivity of the sensors to the scan field rotation described above always adjust the rotation angle for each laser line first to maximise sensor readout. This angle usually doesn’t change much over time unless adjustments have been made to the laser alignment or after the device has been temporarily removed from the objective turret.
3. Although we show that a quite stable output can be achieved over extended periods of time (Fig 6A) it would advisable to double-check with a calibrated power meter at regular intervals.

As biological sample we used standard Alexa488-phalloidin and MitoTracker-red labelled cells (FluoCells Slide #1, ThermoFisher), imaged on a Leica SP5 confocal with a 20x/0.7NA Plan-Apo objective lens, HyD detector, 1400Hz scan speed and pinhole set to 3 airy units to minimise photobleaching as the same sample had to be imaged repeatedly. First we imaged the sample at a typical setting of 5% AOTF (488nm line) at 20% Argon laser power level to adjust the detector gain (Fig 4A). Then the IntensityCheck device was used to obtain the corresponding target intensity value. To emulate fluctuations in laser power we subsequently varied the Argon laser power level (0–100%), which controls the actual light output from the laser head. At each power level IntensityCheck was used first to reach the target value by adjusting the AOTF. Then we switched back to the 20x imaging lens and recorded the sample image. As control we recorded a corresponding image series where the AOTF setting was maintained throughout at 5% (fixed AOTF in Fig 4A). For quantification purposes the mean image intensity was calculated.

A similar experiment was carried out with the laser power meter sensor mounted on the microscope stage using 10x/0.4NA Plan-Apo objective, here we started by measuring 18.8 μW with the meter at 20% laser power/10% AOTF 488nm, followed by 5 rounds of ramping the laser output between 0 and 100%, always using IntensityCheck first to adjust the AOTF settings to reach the target value and then recording the corresponding power meter readings (Fig 4C). For the long-term experiments (Fig 6A) we obtained a target value corresponding to 30 μW/488nm on day one and on subsequent measurement days we ramped Argon laser power levels again between 0%-100%, adjusting the AOTF with IntensityCheck first followed by triplicate laser intensity measurements.

### Measuring day-to-day variability

To examine the extent of daily variation on illumination intensity in routine confocal practice we imaged the same cells of the Fluocells sample repeatedly over a number of days, first thing in the morning (AM) as well as 4 to 6 hours later in the afternoon (PM), depending on system availability (Objective: HCX PL APO CS 10.0x/NA0.40; Zoom 6.0, pinhole 3AU, 4x line averaging). We compared images recorded with the fixed 5% AOTF settings for both the 488nm and 561nm with the IntensityCheck corrected images taken at the same time. Mean image intensities were compared relative to the intensities measured on day one.

## Supporting information

S1 Fig Redesigned IntensityCheck light sensor unit with easy battery access. (A) The new battery holder improves battery replacement. The old battery is simply pulled out, and a new one inserted; the device remains mounted on the objective turret. (B) Bar chart showing the IntensityCheck confocal laser measurements after changing the battery ten times on the redesigned unit (mean intensity and standard deviation; n = 10). Laser power was adjusted to 20% AOTF (20% Argon laser power level), except for the much weaker 594nm laser (50%).
(TIF)

S2 Fig Short-term laser stability and noise. (A) If a power meter with recording function is not available laser stability and noise can simply be measured using the confocal transmitted light detector, here showing very small intensity fluctuations of a 405nm diode laser. (B,C) Subsequent switching to the different IntensityCheck sensor devices (objective shaped sensor mounted on objective turret/slide shaped sensor) reveals similar oscillations indicating the usefulness of the sensors for this purpose. (D) Final transmitted light recording with ongoing oscillations.
(TIF)

S3 Fig Laser power measurements on a TIRF microscope. (A) With an OD 4 neutral density filter mounted in front of the IntensityCheck sensor high laser powers measurements can be taken as well demonstrating the use for routine performance checks. The IntensityCheck readings were maximised at each time-point by steering the 462nm laser beam in the back focal plane onto the small sensor area. (B) Following calibration against a laser power meter on day 1 with IntensityCheck the target intensities (for the 462nm laser) were maintained over time. Shown are triplicate attempts to reach the different target values (50–200mW).
(TIF)

S4 Fig Using IntensityCheck with non-coherent light sources and for alignment purposes. (A) Using IntensityCheck to compare the kinetics of the light output from different conventional fluorescence light sources immediately after switching on. HBO refers to a 100W short-arc mercury bulb.
(B) Testing the effect of temperature on the sensor output. The IntensityCheck unit was mounted on the objective turret of an inverted microscope with a temperature probe inserted into the device to monitor temperature. The microscope was enclosed with an environmental chamber for live cell imaging and at the indicated time-point the heater was turned on. Despite the 10–15°C rise in temperature the sensor readings continued to oscillate around the same level indicating that the sensor itself is not significantly affected by temperature. These small oscillations were due to the room air conditioning unit; raising the set temperature to turn off the cooling (‘aircon off’) led to an increase in room temperature and a small decrease in light intensity, possibly by affecting directly the LED unit and/or the light guide connection with the microscope. Turning the cooling back on reversed those changes.
(C) The alignment of the epi-fluorescence illumination can also be aided by IntensityCheck. Comparison of the light intensities measured with IntensityCheck in the back focal plane with the corresponding images of a fluorescent plastic slide (yellow-green Chroma slide; 10x Plan-Fluorite objective lens) at different settings of the collector lens of a short-arc fluorescence light source. The highest intensities correspond to the most homogeneous field illumination of the sample (false colour representation).
(TIF)

S5 Fig Characterisation of the LED mounted on the colour sensor board. (A) Emission spectrum of the white LED mounted on the TCS34725 colour sensor board as determined with a confocal spectral detector. (B) Kinetics of the LED output after switching it on, showing very stable and repeatable output when powered by the 3V supply of the USB programming module (connected to the USB port of a PC). The light output was measured with the colour light sensor itself as a reflective OD1 neutral density filter was mounted in front of the sensor (average and standard deviation of 8 independent experiments using the same sensor device on different confocal microscopes). The inset shows the rapid decline in light output when using the internal 3V battery of the original sensor design. (C) The images recorded on the different confocal microscopes tested show a very homogeneous field illumination; note the strong contrast enhancement in the pseudocolour representation. The intensity profiles in (D) were measured across the diagonal line shown in (C), with an average of ~180, the target setting for each detector.
(TIF)

S1 Video Demonstrating some of the functionality of the IntensityCheck sensor and app when monitoring laser power on an inverted confocal microscope. After switching on the light detector the app is started and connects via Bluetooth. Most features are shown using the TSL2561 visible/IR light sensor; the end of the video sequence shows the display when using the colour sensor, which can distinguish between the different laser lines.
(MP4)

S1 Text Detailed instructions on the programming, assembly and use of the IntensityCheck sensors and app. (DOCX)

S1 File ZIP archive containing the 3D printer design files in the SketchUp software format and in STL format. The files are required for changing the design of the 3D printed parts and for importing into typical 3D printer software packages.
(ZIP)

S2 File ZIP archive containing the Arduino source and hex code required for programming the RFduino microcontroller. (ZIP)

S3 File ZIP archive containing the source code files required for changing or compiling the IntensityCheck app as described in the S1 Text file. (ZIP)

S4 File ZIP archive containing the compiled IntensityCheck Android app. The installation of the app and how to use it is described in the S1 Text
(ZIP)

## References

[1] Swedlow JR (2013) Quantitative fluorescence microscopy and image deconvolution. Methods in cell biology 114: 407–426. 10.1016/B978-0-12-407761-4.00017-8 23931516

[2] Amos WB, White JG (2003) How the confocal laser scanning microscope entered biological research. Biology of the cell 95: 335–342. 14519550 10.1016/s0248-4900(03)00078-9

[3] Zucker RM (2014) Evaluating confocal microscopy system performance. Methods in molecular biology 1075: 321–374. 10.1007/978-1-60761-847-8_17 24052361

[4] Hng KI, Dormann D (2013) ConfocalCheck—a software tool for the automated monitoring of confocal microscope performance. PloS one 8: e79879 10.1371/journal.pone.0079879 24224017 PMC3818239

[5] Garsha K (2008) Quantitative Fluorescence Microscopy: Considerations and Controls; Wolfbeis OS, editor. Berlin Heidelberg: Springer.

[6] Jonkman J, Brown CM, Cole RW (2014) Quantitative confocal microscopy: beyond a pretty picture. Methods in cell biology 123: 113–134. 10.1016/B978-0-12-420138-5.00007-0 24974025

[7] Deagle RC, Wee TE, Brown CM (2017) Reproducibility in light microscopy: Maintenance, standards and SOPs. The international journal of biochemistry & cell biology 89: 120–124. 28606390 10.1016/j.biocel.2017.06.008

[8] Zucker RM (2006) Quality assessment of confocal microscopy slide-based systems: instability. Cytometry Part A: the journal of the International Society for Analytical Cytology 69: 677–690. 16807898 10.1002/cyto.a.20313

[9] Zucker RM (2006) Quality assessment of confocal microscopy slide based systems: performance. Cytometry Part A: the journal of the International Society for Analytical Cytology 69: 659–676. 16807897 10.1002/cyto.a.20314

[10] Zucker RM, Chua M (2010) Evaluation and purchase of confocal microscopes: numerous factors to consider. Current protocols in cytometry / editorial board, J Paul Robinson, managing editor [et al] Chapter 2: Unit2 16. 10.1002/0471142956.cy0216s54 20938918

[11] Flora Lux Sensor—TSL2561 Light Sensor—v1.0. Available from: https://www.adafruit.com/product/1246

[12] Flora Color Sensor with White Illumination LED—TCS34725. Available from: https://www.adafruit.com/product/1356

[13] van de Linde S, Loschberger A, Klein T, Heidbreder M, Wolter S, Heilemann M, et al (2011) Direct stochastic optical reconstruction microscopy with standard fluorescent probes. Nature protocols 6: 991–1009. 10.1038/nprot.2011.336 21720313

[14] Cho EH, Lockett SJ (2006) Calibration and standardization of the emission light path of confocal microscopes. Journal of microscopy 223: 15–25. 10.1111/j.1365-2818.2006.01598.x 16872427

[15] Russ JC (1994) The Image Processing Handbook: CRC Press, Inc.

