# Supplementary figures and images for: IntensityCheck – The light measuring app for microscope performance checks and consistent fluorescence imaging
Source: PLoS One. 2019 Mar 28;14(3):e0214659. doi: 10.1371/journal.pone.0214659 (PMC6438524; doi:10.1371/journal.pone.0214659)

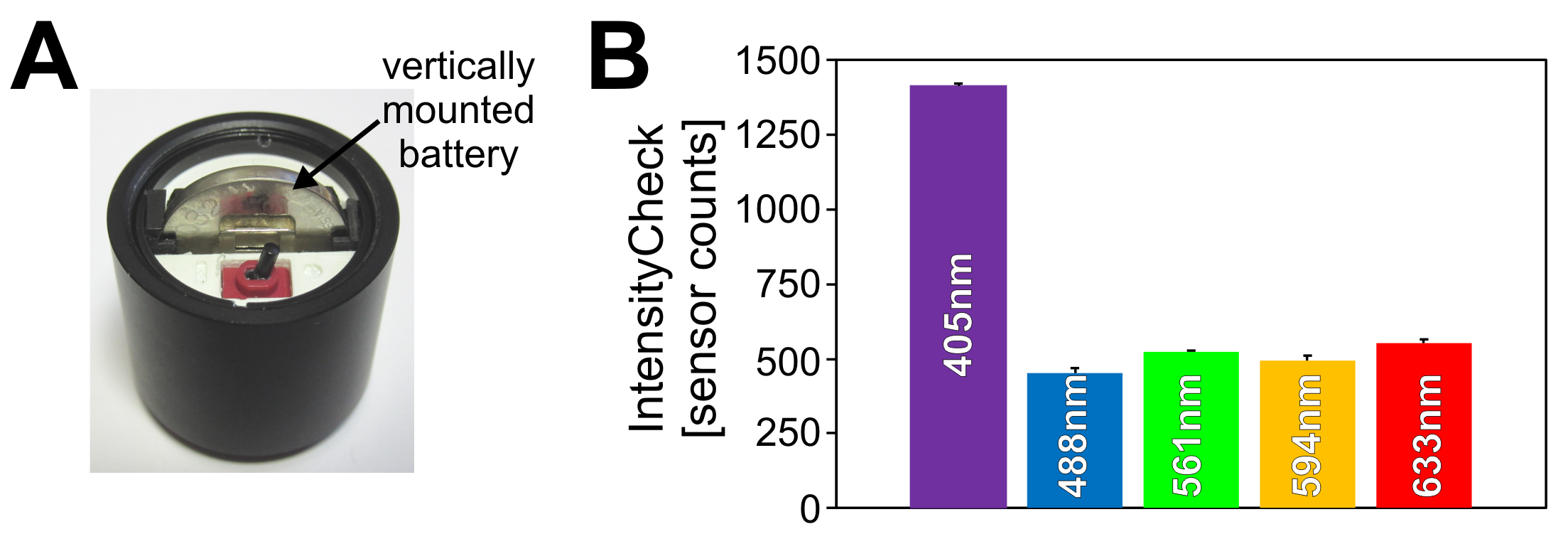

Supplement: S1 Fig — (A) The new battery holder improves battery replacement. The old battery is simply pulled out, and a new one inserted; the device remains mounted on the objective turret. (B) Bar chart showing the IntensityCheck confocal laser measurements after changing the battery ten times on the redesigned unit (mean intensity and standard deviation; n = 10). Laser power was adjusted to 20% AOTF (20% Argon laser power level), except for the much weaker 594nm laser (50%). (TIF) [file pone.0214659.s001.tif]

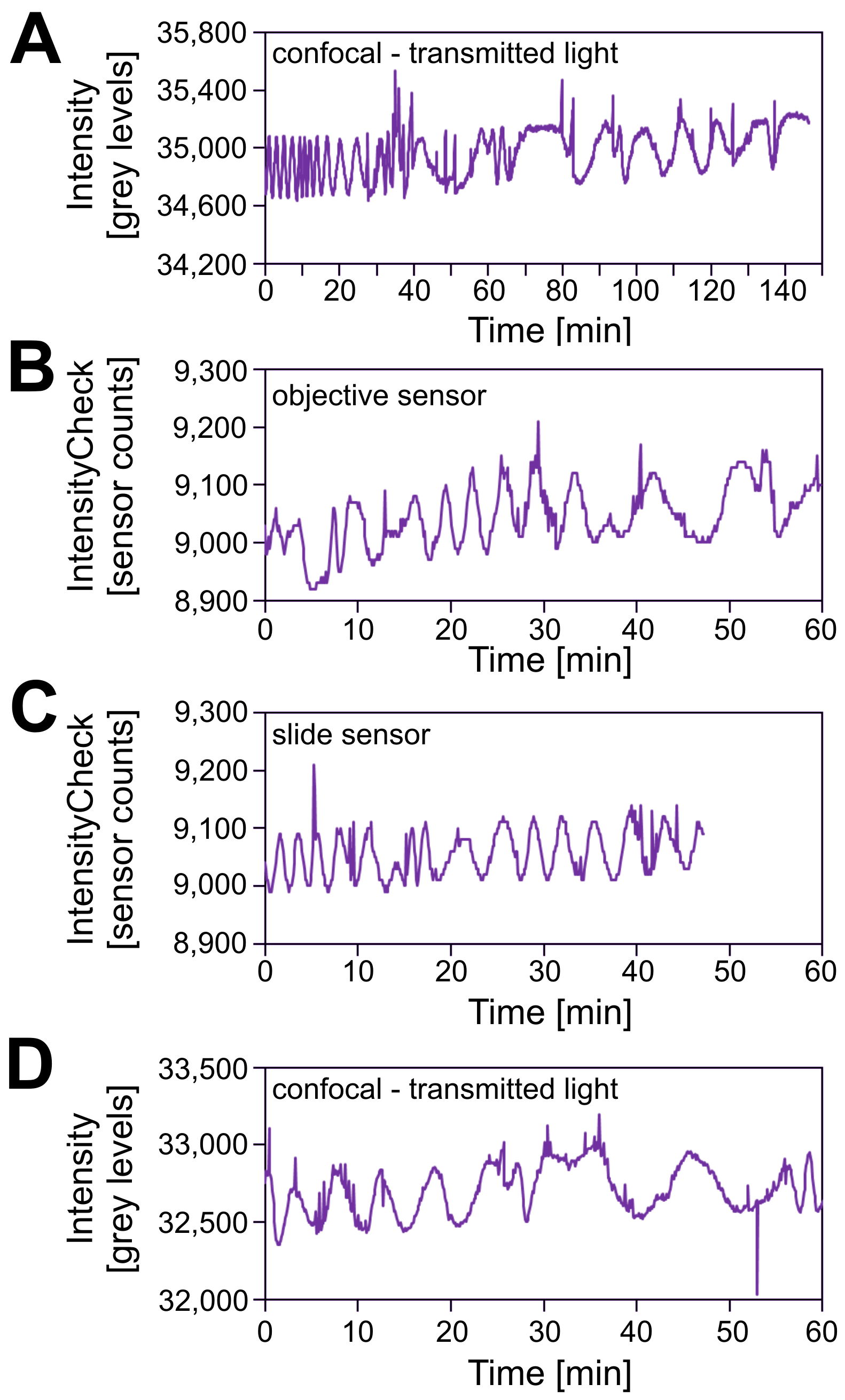

Supplement: S2 Fig — (A) If a power meter with recording function is not available laser stability and noise can simply be measured using the confocal transmitted light detector, here showing very small intensity fluctuations of a 405nm diode laser. (B,C) Subsequent switching to the different IntensityCheck sensor devices (objective shaped sensor mounted on objective turret/slide shaped sensor) reveals similar oscillations indicating the usefulness of the sensors for this purpose. (D) Final transmitted light recording with ongoing oscillations. (TIF) [file pone.0214659.s002.tif]

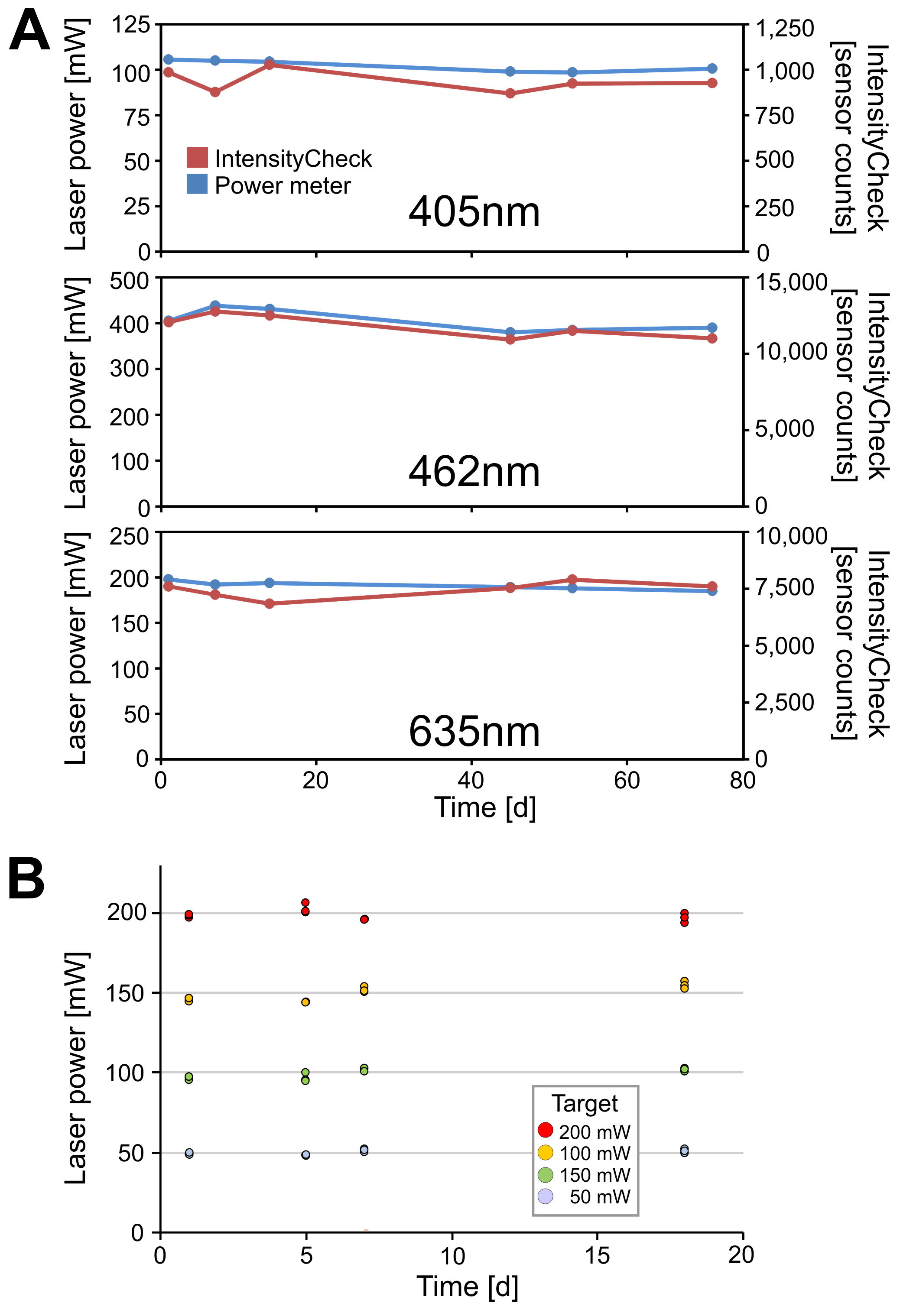

Supplement: S3 Fig — (A) With an OD 4 neutral density filter mounted in front of the IntensityCheck sensor high laser powers measurements can be taken as well demonstrating the use for routine performance checks. The IntensityCheck readings were maximised at each time-point by steering the 462nm laser beam in the back focal plane onto the small sensor area. (B) Following calibration against a laser power meter on day 1 with IntensityCheck the target intensities (for the 462nm laser) were maintained over time. Shown are triplicate attempts to reach the different target values (50–200mW). (TIF) [file pone.0214659.s003.tif]

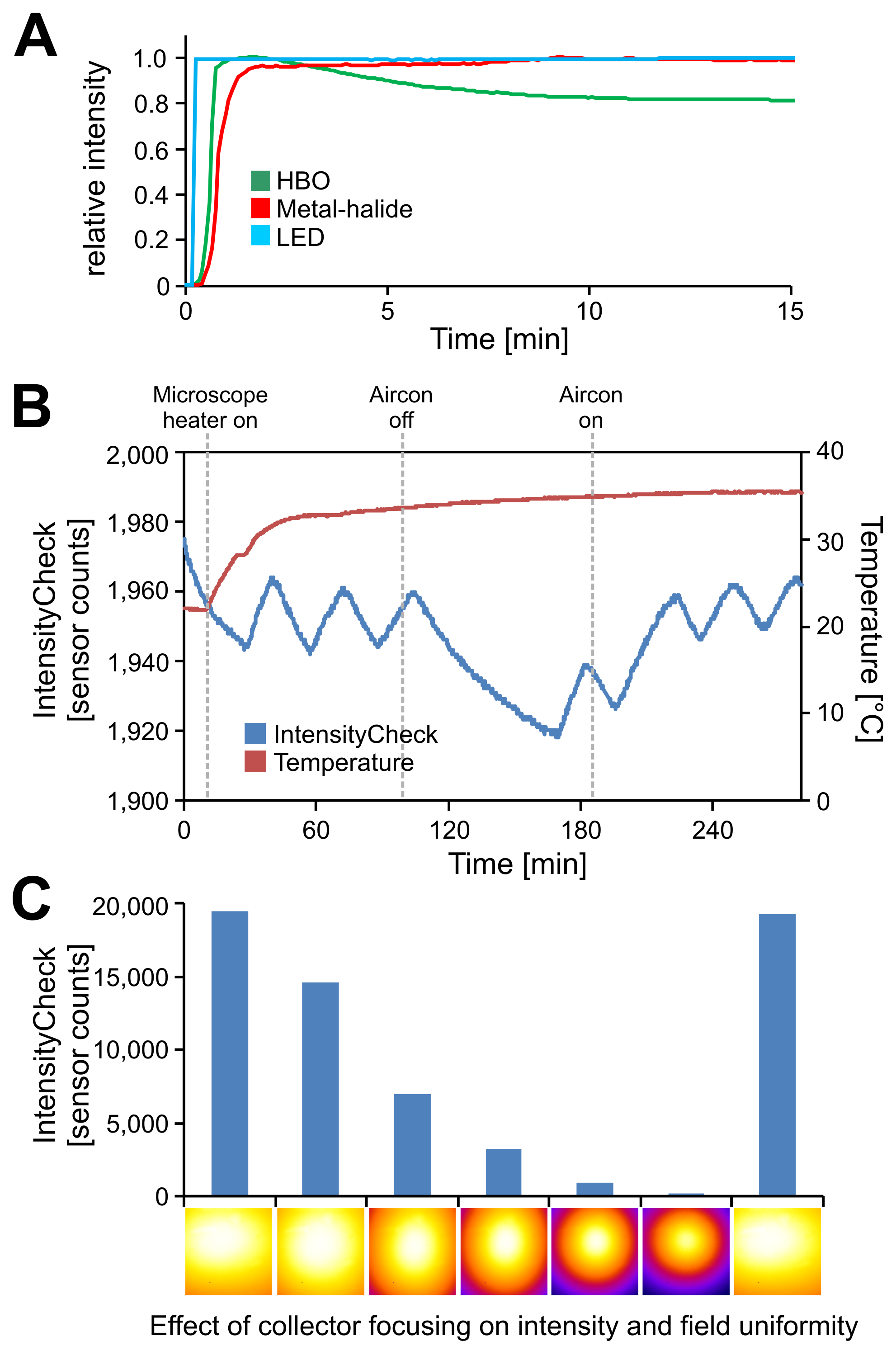

Supplement: S4 Fig — (A) Using IntensityCheck to compare the kinetics of the light output from different conventional fluorescence light sources immediately after switching on. HBO refers to a 100W short-arc mercury bulb. (B) Testing the effect of temperature on the sensor output. The IntensityCheck unit was mounted on the objective turret of an inverted microscope with a temperature probe inserted into the device to monitor temperature. The microscope was enclosed with an environmental chamber for live cell imaging and at the indicated time-point the heater was turned on. Despite the 10–15°C rise in temperature the sensor readings continued to oscillate around the same level indicating that the sensor itself is not significantly affected by temperature. These small oscillations were due to the room air conditioning unit; raising the set temperature to turn off the cooling (‘aircon off’) led to an increase in room temperature and a small decrease in light intensity, possibly by affecting directly the LED unit and/or the light guide connection with the microscope. Turning the cooling back on reversed those changes. (C) The alignment of the epi-fluorescence illumination can also be aided by IntensityCheck. Comparison of the light intensities measured with IntensityCheck in the back focal plane with the corresponding images of a fluorescent plastic slide (yellow-green Chroma slide; 10x Plan-Fluorite objective lens) at different settings of the collector lens of a short-arc fluorescence light source. The highest intensities correspond to the most homogeneous field illumination of the sample (false colour representation). (TIF) [file pone.0214659.s004.tif]

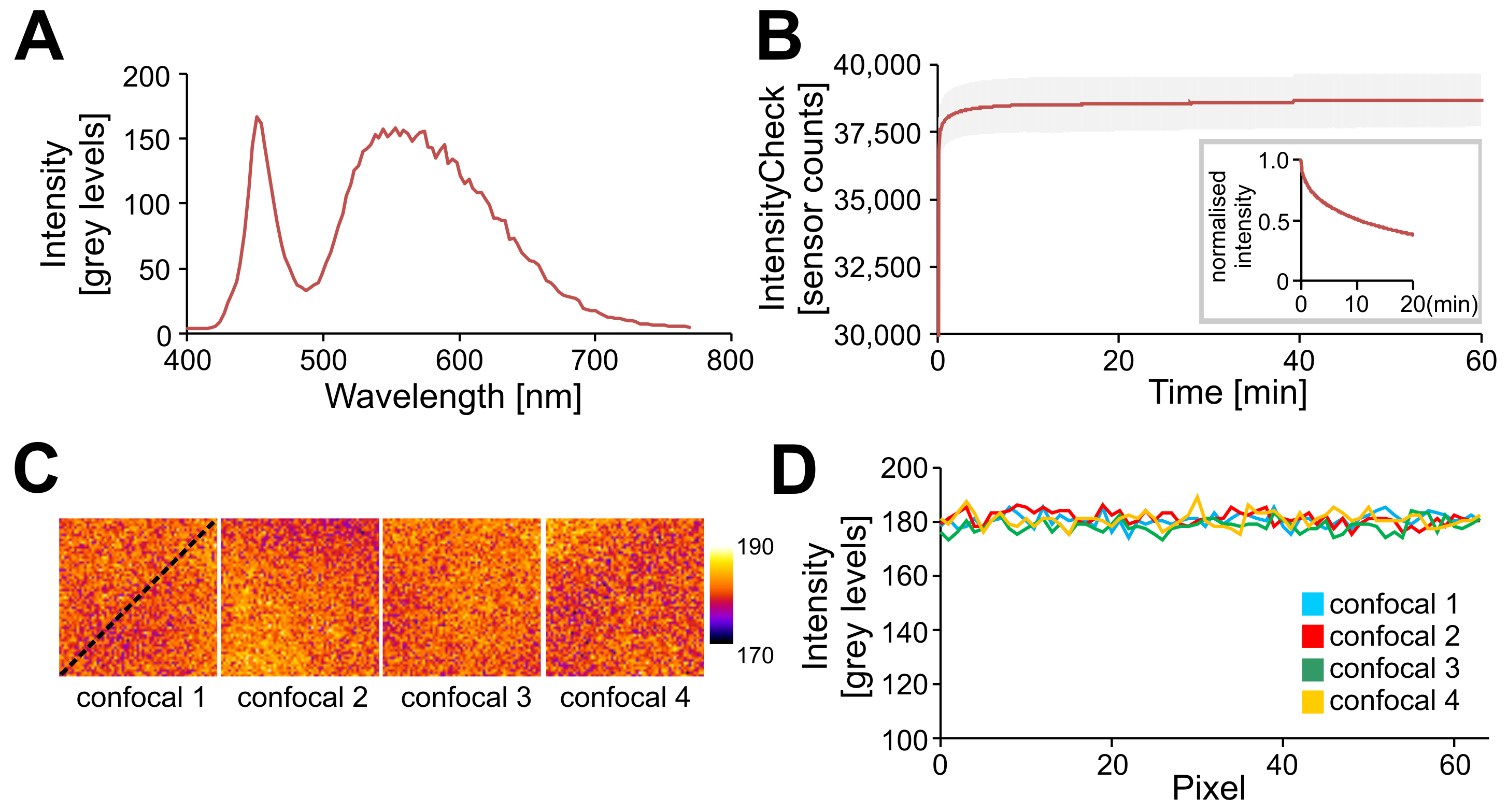

Supplement: S5 Fig — (A) Emission spectrum of the white LED mounted on the TCS34725 colour sensor board as determined with a confocal spectral detector. (B) Kinetics of the LED output after switching it on, showing very stable and repeatable output when powered by the 3V supply of the USB programming module (connected to the USB port of a PC). The light output was measured with the colour light sensor itself as a reflective OD1 neutral density filter was mounted in front of the sensor (average and standard deviation of 8 independent experiments using the same sensor device on different confocal microscopes). The inset shows the rapid decline in light output when using the internal 3V battery of the original sensor design. (C) The images recorded on the different confocal microscopes tested show a very homogeneous field illumination; note the strong contrast enhancement in the pseudocolour representation. The intensity profiles in (D) were measured across the diagonal line shown in (C), with an average of ~180, the target setting for each detector. (TIF) [file pone.0214659.s005.tif]

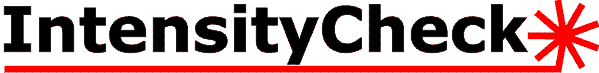

Supplement: S3 File — (ZIP) [file pone.0214659.s010.zip › www/ui/images/IntensityCheck_banner4.png]
