# Supplementary material for: IntensityCheck – The light measuring app for microscope performance checks and consistent fluorescence imaging
Source: PLoS One. 2019 Mar 28;14(3):e0214659. doi: 10.1371/journal.pone.0214659 (PMC6438524; doi:10.1371/journal.pone.0214659)
Supplement: S1 Text — (DOCX) [file pone.0214659.s007.docx]

# **IntensityCheck – the smartphone app and light sensor for microscope performance checks and consistent fluorescence imaging**

**Supporting Text**

Contents

[Introduction 2](#_Toc881586)

[Assembly of the RFduino programming socket 3](#_Toc881587)

[Programming the Rfduino 6](#_Toc881588)

[Assembly of the slide-shaped Light detector 10](#_Toc881589)

[Building the Objective lens-shaped Light detector (version 1) 13](#_Toc881590)

[Assembly into the lens tube holder 17](#_Toc881591)

[Building the improved Light detector (version 2) 18](#_Toc881592)

[The IntensityCheck Android App 25](#_Toc881593)

# Introduction

This document contains detailed instructions on how to build and assemble the IntensityCheck light sensors and how to operate the software.

While the basic electronic circuit is quite simple, due to the small size of the electronic components and the size restrictions of such a compact unit the assembly could be challenging.

Basic soldering experience would be useful but there are many online tutorials available to help (for example: <https://learn.adafruit.com/adfruit-guide-excellent-soldering/>).

A low magnification stereo microscope (or any other suitable magnifying device) is essential for working with the miniature microcontroller.

This guide is split into a number of sections. First we describe the assembly of a programming socket which is useful when multiple microcontroller modules need to be programmed, followed by the programming instructions to load the software onto the Rfduino microcontroller.

Then we describe the assembly of the different sensor units we developed and tested. While most of the experimental data described in the paper were conducted with the objective lens-shaped unit v1 we realised later that the assembly of those units and battery replacement were awkward. We therefore developed an improved version, v2, which simplifies construction and batteries can now easily be exchanged.

Finally we describe how to install and use the Android app that controls the sensor unit and displays the light intensity readings.

# Assembly of the RFduino programming socket

Before the electronic parts can be assembled the RFduino microcontroller needs to be programmed. The RFduino programming socket described here is useful if several RFduino modules need to be programmed as hand soldering of the small contacts on the module is tedious and time consuming. Furthermore different connections on the Rfduino board are required for programming and operation. The parts are easy to source and assemble, however two 3D printed parts are required.

If you don’t want use this device, directly solder the Rfduino programming pins (GPIO0, GPIO1, Reset, +, GND, see page 12 of the RFduino datasheet, <http://www.rfduino.com/documentation/index.html>) to the ribbon cable, solder the other cable ends to individual pins and connect to the USB programming module (Fig 1F,G).

**Required parts** (Fig 1A)

- RFD22121 USB programming module
- 2 Headers (e.g. MULTICOMP 2206RPA-10G, Wire-To-Board Connector, Right Angle, 2206RPA Series, 10 Contacts, Header, 1.27 mm, Through Hole)
- 1 8-pin Arduino shield stacking header (e.g. Adafruit Shield stacking headers for Arduino (R3 Compatible) PRODUCT ID: 85, [https://www.adafruit.com/product/85](https://www.adafruit.com/product/85%20) ).
- 5 pins extracted from Arduino shield stacking header: simply pull out individual metal pins from a stacking header using a pair of pliers.
- 10cm colour flat ribbon cable (e.g. RS Pro 10 Way Unscreened Flat Ribbon Cable, 12.7 mm Width, RS Stock No. 214-0661, <http://uk.rs-online.com/web/p/flat-ribbon-cable/2140661/>)
- 1 screw (~15mm long, 2mm diameter) with 3 matching washers and nut.
- 3D printed parts:
  - Design file created with free Sketchup Make 2016 software (<http://www.sketchup.com/>): RfDuino_Programmer_v1-0_14082018.skp
  - RfDuino_Programmer_top_part_v1-0_14082018.stl
  - RfDuino_Programmer_bottom_part_v1-0_14082018.stl

**Assembly**

- Place the rectangular 10-way headers in the 3D printed part as shown and the washer over the hole (Fig 1B). Slightly bend the five pins required for programming upwards (labelled in Fig 1B).
- Put on the lid, place one washer over the hole and insert the screw (Fig 1C). Turn the unit around, place the last washer over the screw and tighten with the nut (Fig 1D).
- Put the unit upside down and bend some of the header pins sideways to prevent the headers from falling off (Fig 1E). Solder the flat ribbon cable to the pins as indicated, this will connect the relevant contacts for programming (see page 12 in rfduino.datasheet.pdf, available from <http://www.rfduino.com/documentation/index.html>).
- Solder the other end of the cables to the 5 individual header pins and push the pins into the appropriate position in the 8-pin stacking header (Fig 1F). By using this additional stacking header – rather than connecting directly to the programming board – handling is improved as one only has to insert/remove the entire header rather than doing it for the five individual pins.
- Insert the stacking header into the USB programming module as shown (Fig 1G).
- Load the Rfduino SMT module into the base with the white antenna facing away from the fixing screw (Fig 1H, see arrow).
- Rotate the lid over the RFduino and tighten the screws to provide a tight connection between the programmer and the RFduino (Fig 1I).
- Now follow the steps described below in *Programming the Rfduino*.

**Troubleshooting**

Care has to be taken when soldering the connections on the headers pins, which are very close together. If a problem occurs during the programming of the RFduino (error message: ‘Timeout reading from uart’), this can be due to poor connections between the RFduino and the header pins in the programmer base. To improve the connectivity slightly bend the five pins required for programming (Fig. 1B) upwards and repeat the programming.

**Fig. 1 Assembly of the RFduino programming socket.** (next page)

(**A**) Parts required for assembly. (**B**) – (**F**) Sequential assembly of the parts. (**G**) Connecting to the USB adapter board. (**H**) Placing the RFduino in the unit for programming. (**I**) By rotating the lid over the RFduino the unit should be pressed against the metal contacts for electrical conductivity allowing the programming of the unit.


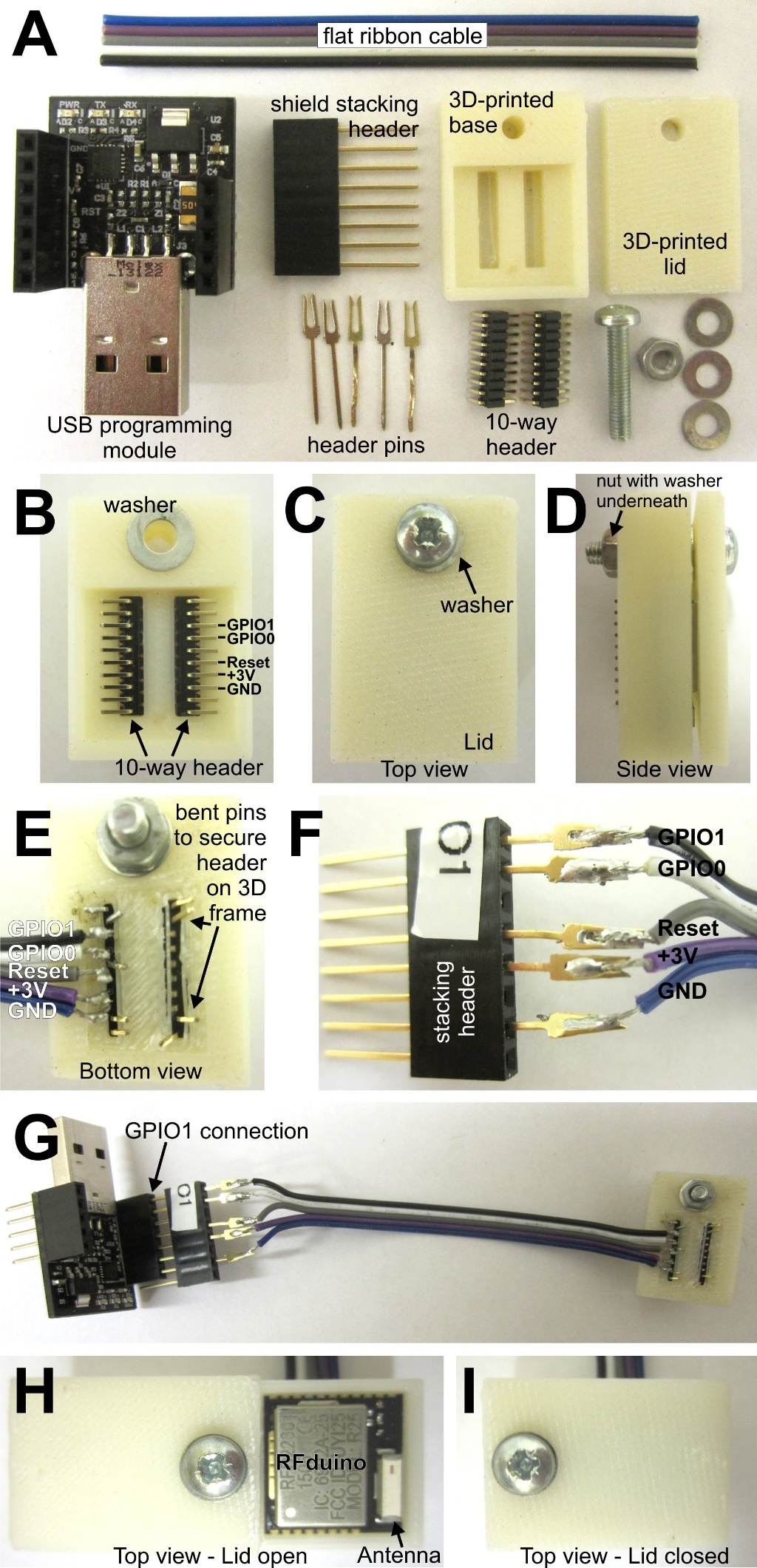


# Programming the Rfduino

Beside the hardware described above to physically connect the Rfduino module to the computer for programming the following software packages are required:

- Arduino Integrated Development Environment (IDE) (<https://www.arduino.cc/en/Main/Software>; we used version 1.8.6 on a Windows 10 PC)

- Install the Rfduino board support for the Arduino IDE (detailed instructions at <https://github.com/RFduino/RFduino/blob/master/README.md> ,

<http://www.rfduino.com/wpcontent/uploads/2014/04/RFduino.Quick_.Start_.Guide_.pdf>)

Essentially, in the Arduino IDE *Preferences*, the link <http://rfduino.com/package_rfduino166_index.json> has to be added in ‘Additional Board Manager URLs’.

Then open the *Boards Manager* in *Tools – Board*, scroll down to **RFduino Boards** and click on *Install* to install the software library (version 2.3.3).


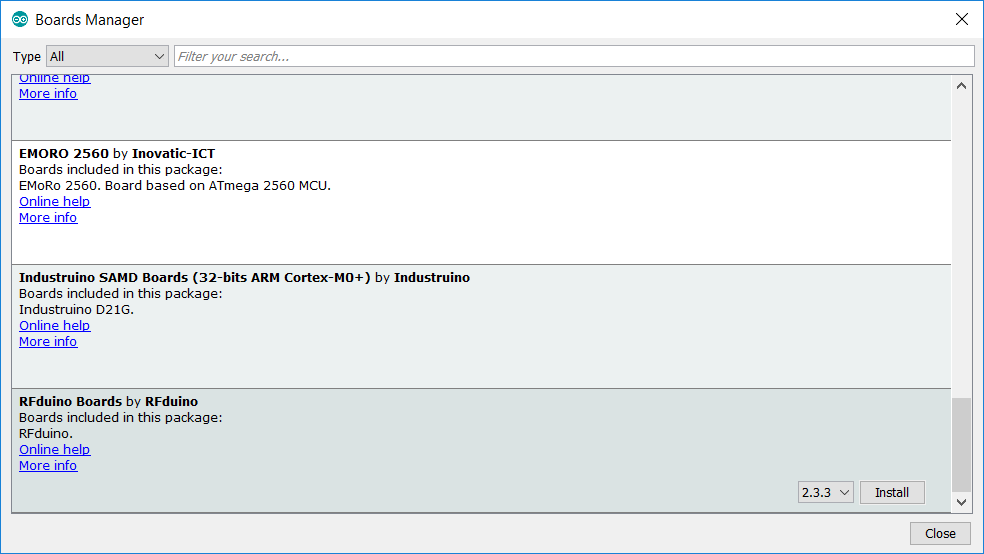


If you want to modify the original source code additional software libraries need to be installed as described below, otherwise continue on page 9:

- Download and install the Adafruit TCS34725 Color Sensor library (<https://github.com/adafruit/Adafruit_TCS34725>) as described in (<https://learn.adafruit.com/adafruit-all-about-arduino-libraries-install-use>).
- Download and install the Adafruit TSL2561 Light Sensor library (<https://github.com/adafruit/Adafruit_TSL2561>):

the file *Adafruit_TSL2561_U.cpp* needs to be modified to allow the automatic detection of the sensor type during startup of the RFduino:

in the function

*boolean Adafruit_TSL2561_Unified::init()* replace: *if (x == 0xFF)* with: *if (x >= 0x1F)*

- Mount the RFduino SMT module (RFD22301) in the programming socket and connect to the USB programming module (RFD22121) as described above (Fig 1G-I).
- Plug the USB module into a free USB port on your computer.
- Run the Arduino IDE software and open the *IntensityCheck_v1-0_14082018.ino* file.
- Modify the source code as required.
- Select the RFduino board and the required COM port (here COM7):


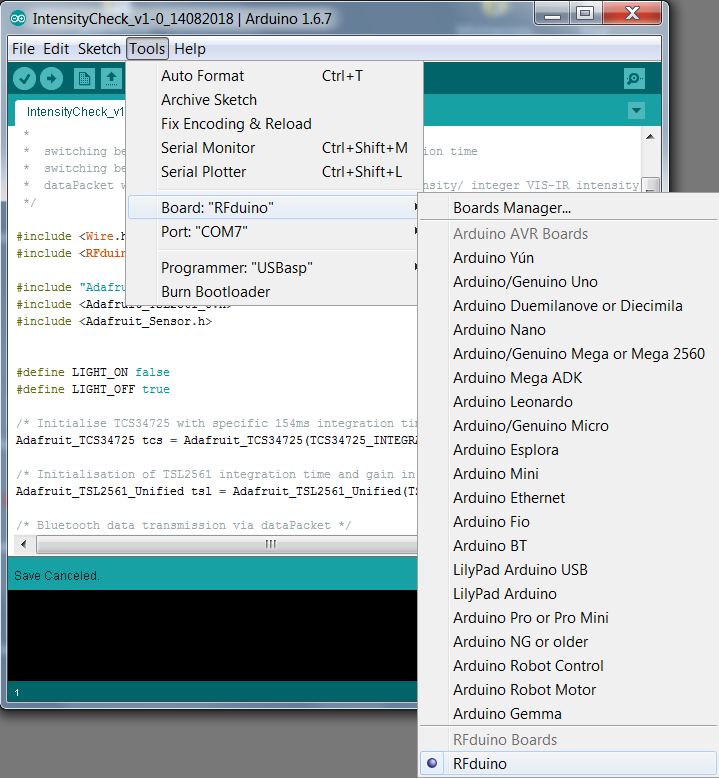


- Select Sketch - Upload. This will compile the program and upload the code to the connected Rfduino module. If the module has been successfully programmed SUCCESS ! will mark the event as shown.


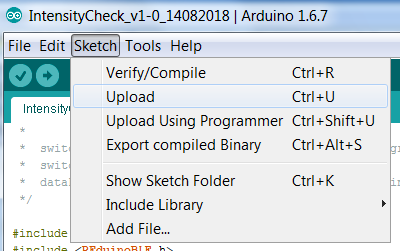

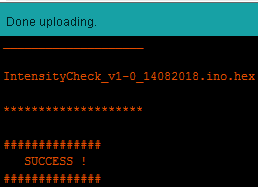


- Test the Rfduino module by selecting the Arduino *Serial Monitor*:


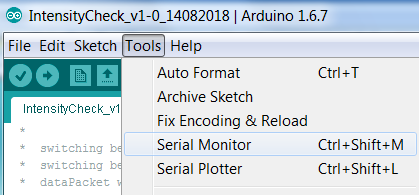


- The following message should appear indicating that the microcontroller is communicating with the PC and looking for a light sensor:


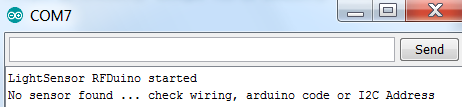


- The Rfduino is now ready for assembly into the various detectors.
- If the programming wasn’t successful (for example error message: ‘Timeout reading from uart’) it could be due to problems with the electrical connections, see troubleshooting section on page 4.

If you don’t need to change the source code and simply want to upload the pre-compiled code to the RFduino, mount the Rfduino module in the programmer and connect to the USB connection as described above (Fig 1G-I):

- Copy the file *IntensityCheck_v1-0_14082018.ino.hex* into the folder containing the *RFDLoader.exe* program, located somewhere in the Arduino Data folder: *..\ArduinoData\packages\RFduino\hardware\RFduino\2.3.3*
- Open the Windows command line window, change to that folder and run the command for the correct USB COM port (here COM7):

*RFDLoader.exe -q COM7 IntensityCheck_v1-0_14082018.ino.hex*


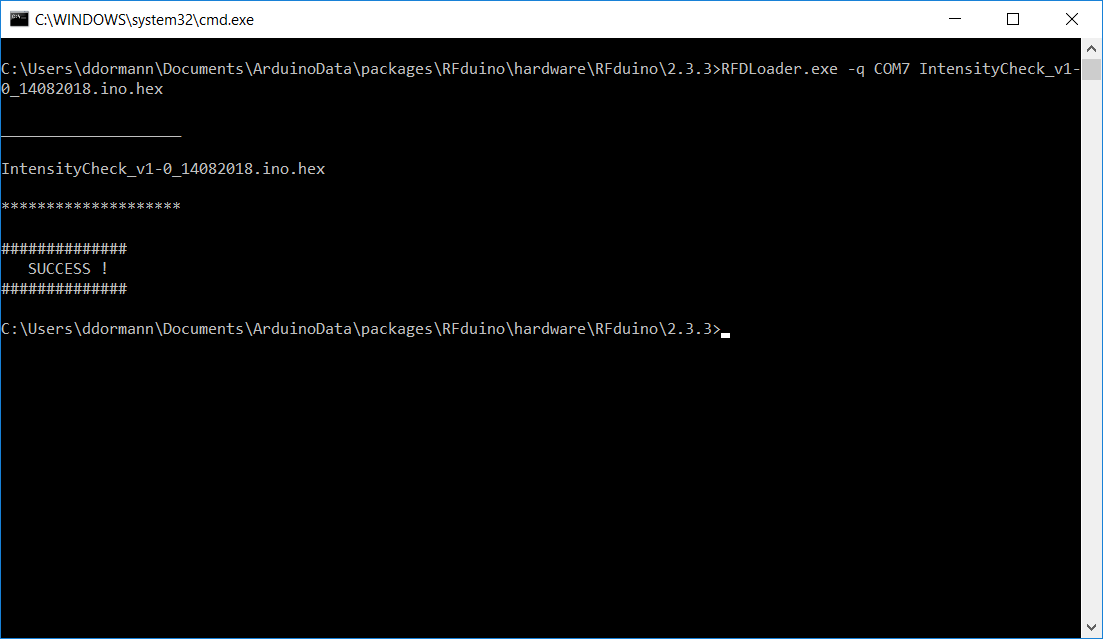


- Test the unit as described above (page 8).

# Assembly of the slide-shaped Light detector

The microscope slide-shaped light detector is the easiest sensor to assemble, however some experience in soldering and handling electronic parts is required. A low magnification stereo microscope (or any other suitable magnifying device) is essential for working with these small electronic components.

**Required parts** (Fig 2A)

- RFD22301 RFduino SMT module (already programmed as described above)
- Flora Colour Sensor with white illumination LED - TCS34725 (Adafruit, Product ID: 1356, <https://www.adafruit.com/product/1356>)

or

Flora Lux Sensor - TSL2561 Light Sensor (Adafruit, Product ID: 1246, <https://www.adafruit.com/product/1246>)

- Ultraminiature toggle switch (e.g. MULTICOMP 2US1T1A1M2RE Toggle Switch, 2US1 Series, Non Illuminated, SPDT, On-On, Through Hole, 100 mA; <http://uk.farnell.com/multicomp/2us1t1a1m2re/switch-ultraminiature-spdt-on/dp/1550199>)
- 10cm colour flat ribbon cable (e.g. RS Pro 10 Way Unscreened Flat Ribbon Cable, 12.7 mm Width; RS Stock No. 214-0661, <http://uk.rs-online.com/web/p/flat-ribbon-cable/2140661/>)
- Copper wire (~0.5mm diameter of the copper core), 3 small pieces to connect to battery
- 3V Battery CR2032
- Neutral density filter (25mm diameter, e.g. Thorlabs ND10B, unmounted N-BK7, OD: 1.0 or higher as required) or glass window (25.4mm diameter x 1mm, e.g. High Efficiency Window, Edmund Optics, stock No. #48-924)
- 6 Neodymium disk magnets (3mm diameter, 2mm thick; e.g. RS Stock No. 434-6827, [http://uk.rs-online.com/web/p/magnets/4346827/](http://uk.rs-online.com/web/p/magnets/4346827/%20) )

- 3D printed parts:
  - Design file created with free Sketchup Make 2016 software (<http://www.sketchup.com/>): SlideSensor_v1-0_14082018.skp
  - SlideSensor_top_part_v1-0_14082018.stl
  - SlideSensor_bottom_part_v1-0_14082018.stl

**Assembly**

- Fix the 6 magnets with super glue in the holes as indicated in Fig 2B, ensure their correct orientation so that the base and the lid are strongly held together.
- Place the switch and light sensor into the base. The colour sensor board shown will just fit in; the diameter of the TSL2561 board is smaller, it should be centred and fixed with ‘super glue’ (Fig 2C). Ensure the orientation of the sensor as shown in the inset (3V connection), the three pins on the switch should face upwards.
- Prepare and bend two pieces of copper wire as shown (Fig 2D insets). These wires should be under tension and protrude from the cut-outs in the printed base so that firm contact is made with the battery. Solder the other ends to the switch (right pin) and the sensor board (GND/ - connection). A third short cable is required to connect the switch (centre pin) to the 3V contact on the sensor board.
- Prepare the colour flat ribbon cables. The yellow and green cable is about 2cm long, the others ~3.7-3.8cm. Solder the short yellow-green cables to the SDA and SCL connections, the longer cables to the 3V and GND contacts (Fig 2D insets).
- Place the RFduino module into the unit, bend and twist the cables as shown (Fig 2D). Note that the neutral density filter will be placed over the sensor unit and it shouldn’t be pushed up by the wires. Carefully solder the contacts on the RFduino.
- Put the neutral density filter and the battery into position (Fig 2E). The reflective filter surface should be facing upwards away from the electronics, the + connection of the battery should face upwards.
- Attach the lid (Fig 2F) and test the assembly by switching on the unit – push the switch to the left. If the colour sensor board is used and all works well the LED on the sensor board will light up briefly (Fig 2G). ). Alternatively run the IntensityCheck App to connect to the sensor or check whether a Bluetooth device called “LightSensor” is present.

**Troubleshooting**

Care has to be taken when soldering the connections in particular on the RFduino unit. If the LED light doesn’t flash when switched on check connections to the battery, the copperwire contacts might have to be pushed out or bend slightly. One could also measure the voltage on the light sensor board (3V vs GND) or the RFduino (contacts of the red vs the brown cable) with a voltmeter to test whether the connections are in order.

**Fig 2. Assembly of the slide-shaped light detector.** (next page)

(**A**) Parts required for assembly. (**B)** – (**F**) Sequential assembly of the parts. (**H**): If the Colour Sensor is used, the LED with flash briefly when switched on, confirming correct assembly.


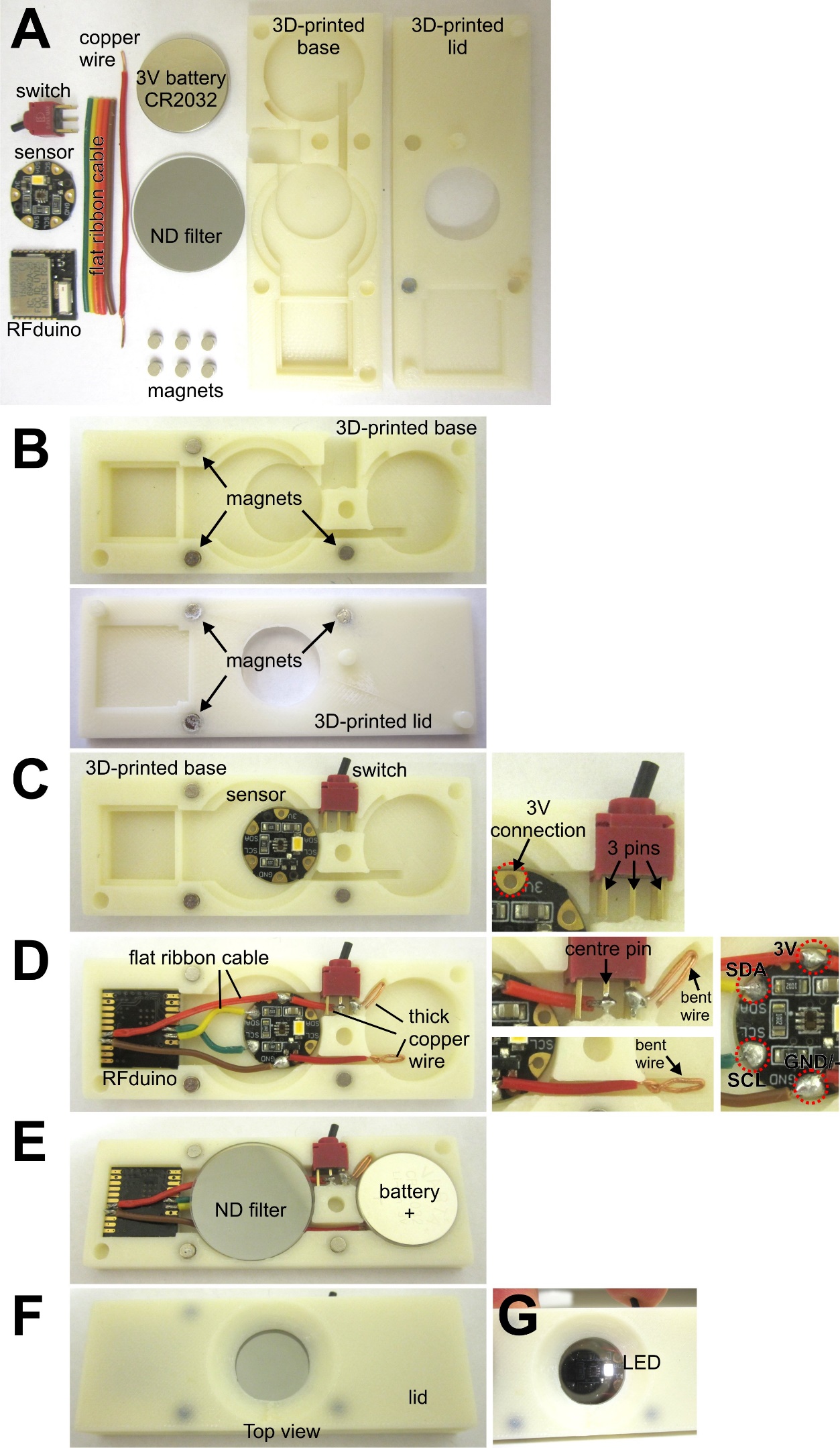


# Building the Objective lens-shaped Light detector (version 1)

The assembly of the objective-shaped light detector is the more demanding than the slide-shaped sensor due to the vertical stacking of the components in the cylindrical 3D printed frames. Although most data in the paper were gathered using this design we have developed an alternative unit (version 2) that should be easier to assemble (page .

Some experience in soldering and assembling electronic parts is required. A low magnification stereo microscope (or any other suitable magnifying device) is essential for working with these small electronic components.

**Required parts** (see Figs 3A and 4A)

- RFD22301 RFduino SMT module (already programmed)
- Flora Colour Sensor with white illumination LED - TCS34725 (Adafruit, Product ID: 1356; <https://www.adafruit.com/product/1356> )

or

Flora Lux Sensor - TSL2561 Light Sensor (Adafruit, Product ID: 1246, <https://www.adafruit.com/product/1246>)

- Ultraminiature toggle switch (e.g. MULTICOMP 2US1T1A1M2RE Toggle Switch, 2US1 Series, Non Illuminated, SPDT, On-On, Through Hole, 100 mA; <http://uk.farnell.com/multicomp/2us1t1a1m2re/switch-ultraminiature-spdt-on/dp/1550199>)
- 10cm colour flat ribbon cable (e.g. RS Pro 10 Way Unscreened Flat Ribbon Cable, 12.7 mm Width; RS Stock No. 214-0661, <http://uk.rs-online.com/web/p/flat-ribbon-cable/2140661/>)
- Copper wire (~0.5mm diameter of the copper core), 2 small pieces to connect to battery
- 3V Battery CR2032
- Neutral density filter (25mm diameter, e.g. Thorlabs ND10B, unmounted N-BK7, OD:1.0 or higher as required) or simple glass window (25.4mm diameter x 1mm, High Efficiency Window, Edmund Optics, stock No. #48-924)
- Thorlabs SM1 Lens Tube, 1.00" (SM1L10)
- Thorlabs Retaining Rings 2× (SM1RR)
- Adapters to mount detector on various microscopes:

| **Microscope Manufacturer** | **Thorlabs part** |
| --- | --- |
| Leica, Nikon | SM1A11 (External M25 x 0.75 Threads and Internal SM1 Threads) |
| Olympus, Zeiss (RMS) | SM1A4 (Adapter with External RMS Threads and Internal SM1 Threads) |
| Zeiss (M27) | SM1A36 (External M27 x 0.75 Threads and Internal SM1 Threads) |

- 3D printed parts:
  - Design file created with free Sketchup Make 2016 software (<http://www.sketchup.com/>): ObjectiveSensor_v1-0_14082018.skp
  - ObjectiveSensor_top_part_v1-0_14082018.stl
  - ObjectiveSensor_bottom_part_v1-0_14082018.stl

**Assembly of the electronic components**

- Cut the cables to roughly the required length as shown in Fig 3B (better too long than too short so that adjustments can be made during assembly and wiring).
- Solder the cables to the sensor as shown (Fig 3C, connections 3V/GND/SDA/SCL).
- Carefully solder the other end of the cables to the Rfduino module (Fig 3D).
- Solder on the small blue cable (Fig 3E, arrow).
- Bend the contacts on the toggle switch as shown (Fig 3F) as they are not required. Alternatively cut them off.
- Solder the blue cable ends to the centre pin of the switch (Fig 3G, blue arrow) and the copper wire (the + battery contact) to the right pin (red arrow).
- As all electronic parts have now been connected the unit needs to be mounted into the 3D printed frame.
- Bend the copper wire connected to the sensor board so that it fits into the groove provided in the 3D frame (see arrow in Fig 3H) when the sensor board is mounted (Fig 3I). The copper wire end should be bend so that the metal will make direct contact with the battery once that is inserted into the central compartment.
- Ensure the sensor board can sit flat on its base (Fig 3I), then fix the board to the base with a drop of ‘super glue’. The 3D frame was originally designed for the larger colour sensor board, when using the smaller TSL2561 circuit board centre the sensor first before fixing with glue. Rotate the RFduino/cable/switch assembly to the other side of the 3D frame (arrow).
- On the other side of the 3D frame carefully push the switch and RFduino into the spaces provided (Fig 3J). Line up the cables and push them down or slightly bend as required to create a neat, tightly packed unit. The 2^nd^ bent copper wire needs to be inserted into its groove (see arrow), ensure the bend or twisted metal end of the wire can make direct contact with the battery. During this final assembly it might be useful to have a battery in the battery compartment to keep the copper wires in position while manipulating other parts.
- If the switch or the Rfduino are too loose they can also be fixed to the 3D frame with small amounts of ‘super glue’.
- Finally insert the 3V battery with the clearly labelled + side of the battery facing the switch/Rfduino side as indicated (Fig. 3J).
- Test the assembly by switching on the unit. If the colour sensor board is used and all works well the LED on the sensor board will light up for about a second (Fig 3K). Alternatively run the IntensityCheck App to connect to the sensor or check whether a Bluetooth device called “LightSensor” is present.

**
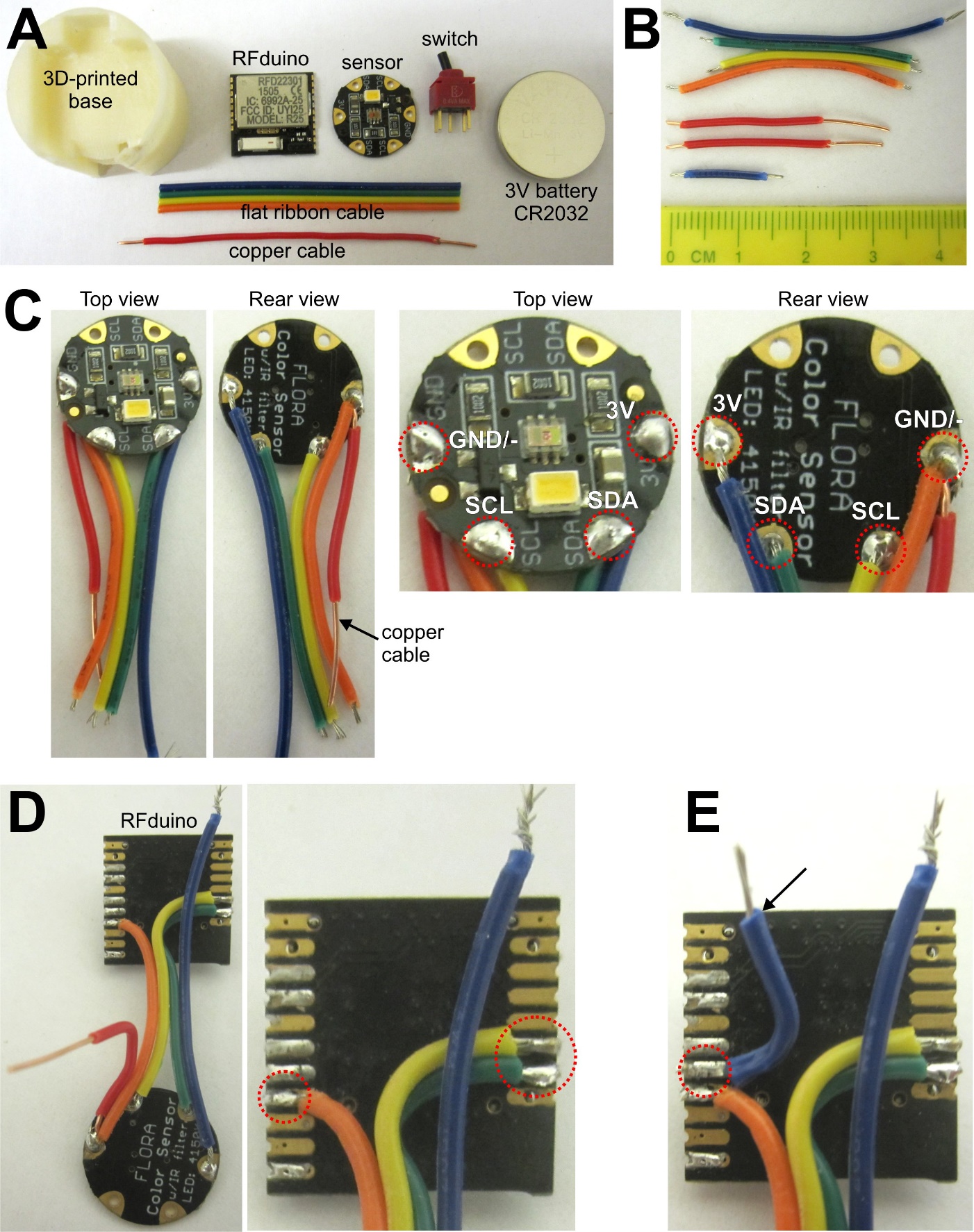
**

**Fig 3. Assembly of the light detector electronics**

(**A**) Parts required for assembly. (**B**) Cable length requirements. (**C**) – (**J**) Sequential assembly of the parts.

**
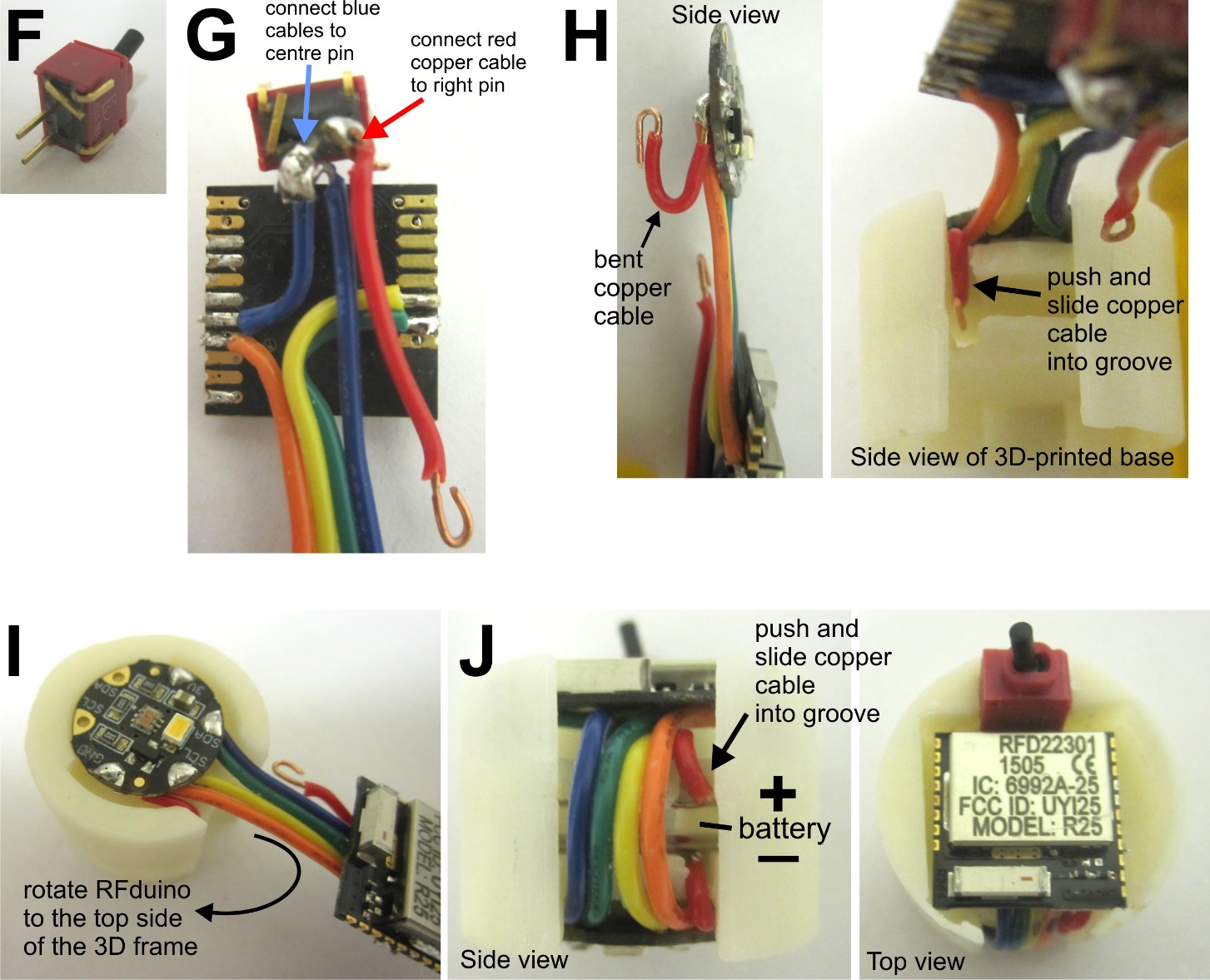
**

**Fig 3. Assembly of the light detector electronics** (continued from previous page)

**Troubleshooting**

Care has to be taken when soldering the connections in particular on the RFduino unit. If the LED light doesn’t flash when switched on or when the Bluetooth device ‘LightSensor’ is not found check connections to the battery, the copperwire contacts might have to be pushed out or bend slightly.

# Assembly into the lens tube holder

- Fig 4A shows the parts required for the final assembly of the unit.
- Insert the neutral density filter with the reflective side facing away from the lens tube and secure with retaining ring (Fig 4B).
- Place the detector unit in the lens tube (Fig 4C), the unit will rest on the retaining ring!
- Put on the 3D printed lid and secure with 2^nd^ retaining ring (Fig 4D).
- Choose appropriate microscope adapter (see page 13) and screw onto the lens tube.


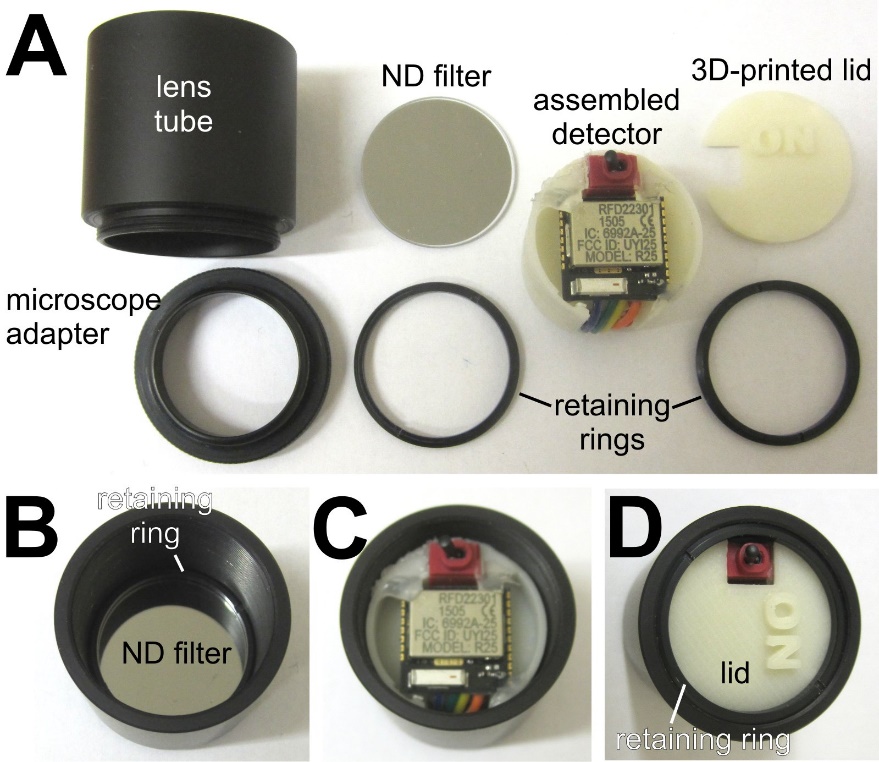


**Fig 4. Mounting the light detector electronics.**

(**A**): Parts required for assembly. (**B**)– (**D**) Sequential assembly of the parts.

Building the improved Light detector (version 2)

In this improved design most connections are already soldered before the assembly into the 3D printed base. These pre-assembled parts are then sequentially inserted into the 3D frame, only a few individual wires need to be aligned and soldered. The 3D frame is specifically designed for the TSL2561 light sensor which provides a better performance than the colour sensor.

Some experience in soldering and assembling electronic parts is required. A low magnification stereo microscope (or any other suitable magnifying device) is essential for working with these small electronic components.

**Required parts** (see Figs 4A and 5A)

- RFD22301 RFduino SMT module (already programmed)
- Flora Lux Sensor - TSL2561 Light Sensor (Adafruit, Product ID: 1246, <https://www.adafruit.com/product/1246>)
- Ultraminiature toggle switch (e.g. MULTICOMP 2US1T1A1M2RE Toggle Switch, 2US1 Series, Non Illuminated, SPDT, On-On, Through Hole, 100 mA; <http://uk.farnell.com/multicomp/2us1t1a1m2re/switch-ultraminiature-spdt-on/dp/1550199>)
- 10cm colour flat ribbon cable (e.g. RS Pro 10 Way Unscreened Flat Ribbon Cable, 12.7 mm Width; RS Stock No. 214-0661, <http://uk.rs-online.com/web/p/flat-ribbon-cable/2140661/>). Individual cable lengths as shown in Fig 5A .
- Copper wire (~0.5mm diameter of the copper core), 2 small pieces to connect to battery
- Vertical battery holder (HARWIN Coin Cell Battery Holder, S8401-46; <https://uk.rs-online.com/web/p/battery-holders-mounts/1613608/>)
- 3V Battery CR2032
- 3D printed parts:
  - Design file created with free Sketchup Make 2016 software (<http://www.sketchup.com/>): ObjectiveSensor_v2-0_12022019.skp
  - ObjectiveSensor_v2-0_12022019.stl
- Neutral density filter (25mm diameter, e.g. Thorlabs ND10B, unmounted N-BK7, OD:1.0 or higher as required) or simple glass window (25.4mm diameter x 1mm, High Efficiency Window, Edmund Optics, stock No. #48-924)
- Thorlabs SM1 Lens Tube, 1.00" (SM1L10)
- Thorlabs Retaining Rings 2× (SM1RR)
- Adapters to mount detector on various microscopes:

| **Microscope Manufacturer** | **Thorlabs part** |
| --- | --- |
| Leica, Nikon | SM1A11 (External M25 x 0.75 Threads and Internal SM1 Threads) |
| Olympus, Zeiss (RMS) | SM1A4 (Adapter with External RMS Threads and Internal SM1 Threads) |
| Zeiss (M27) | SM1A36 (External M27 x 0.75 Threads and Internal SM1 Threads) |

**Assembly of the electronic components**

- Cut the cables to the required lengths as indicated in Fig 5A. We will assemble the three units (battery assembly holder, switch, RFduino) separately first (Fig 5B-E).
- Preparation of the battery holder.
  - Cut off a small part from one side of the battery holder as indicated by the red circles (Fig 5B). This is necessary as otherwise it will not be possible to remove the battery from the finished mounted sensor unit.
  - Solder the cables to the contacts as indicated (Fig 5C). For alignment during assembly it is best to remove the insulation from the copper wire.
- Preparation of the switch:
  - Solder the grey and white cables to the right pin of the switch (Fig 5D).
- Preparation of the RFduino:
  - Carefully solder the green and yellow cables Rfduino module (Fig 5E).


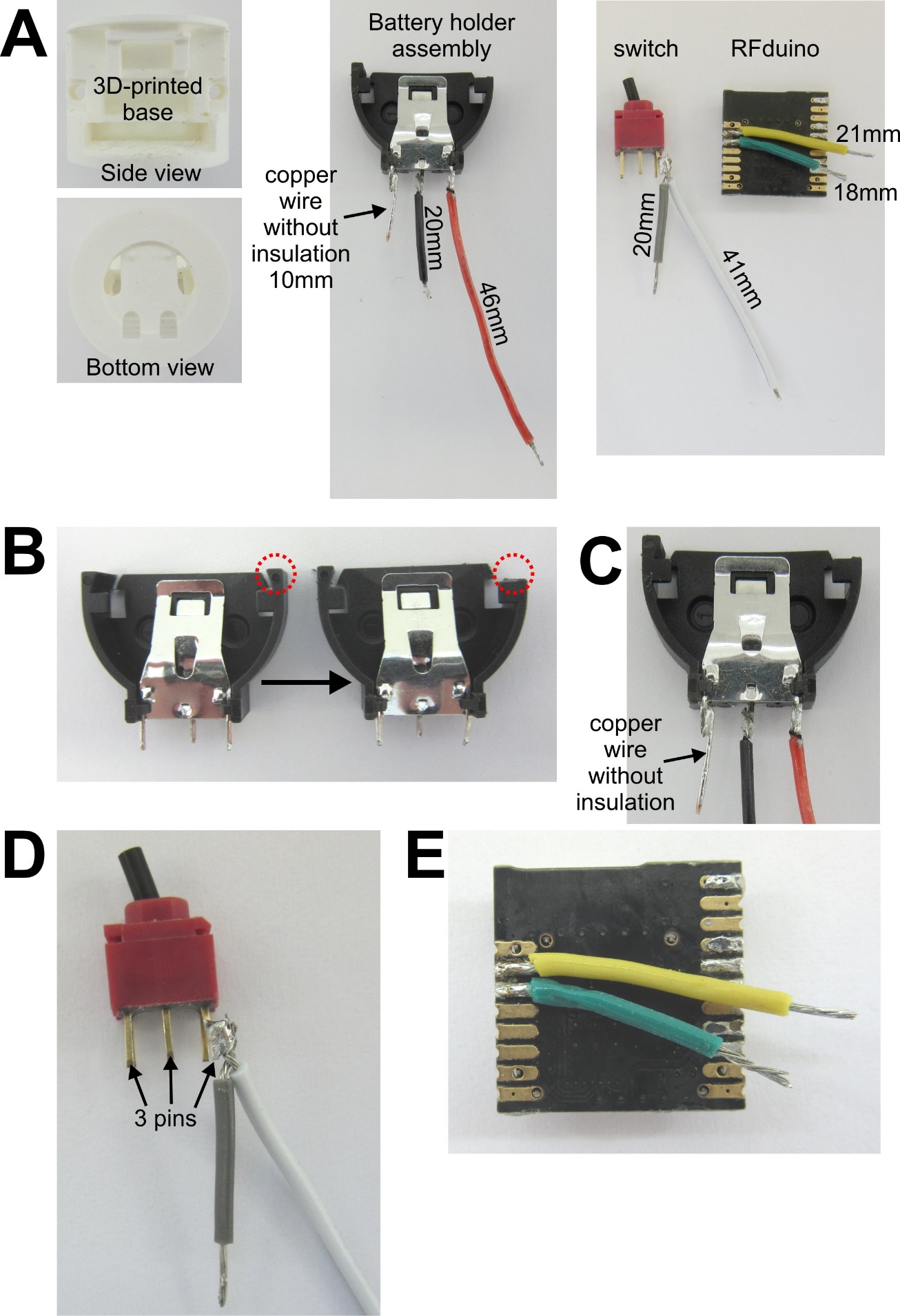


**Fig 5. Assembly of the main parts for the light detector electronics v2.**

(**A**) Parts required for assembly. (**B-E**) Assembly of the main components.

- With the main components prepared we can start to mount them on the 3D printed frame:
- Push the TSL2561 sensor board into position on the bottom side of the 3D frame (Fig 6A). Align the board so that the holes of the SDA and SCL connections on the board fit over the two protrusions (marked by the two arrows). A few very small drops of superglue applied to the edges of the sensor board – but away from the other soldering contacts – help to keep the sensor in place.
- Take the pre-assembled battery holder and move it slowly into the central cavity of the 3D frame, with the metal clip marked “+” facing the viewer (Fig 6B, red circle). Align the copper wire as indicated as that should be pushed through the hole of the 3V connection on the sensor board (black arrows in Fig 6B,D). Pull the cables carefully around as shown.
- Push the battery holder down as far as possible as indicated in Fig 6C. Fix the battery holder onto the 3D frame with superglue but avoid getting any glue on the electrical contacts.
- Turn the unit around to view the sensor board, the copper wire should be protruding now through the 3V connection (Fig 6D).
- Solder the 3V connection (red circle in Fig 6E) and cut off any protruding wire.
- Slide the switch slowly into the 3D frame with the 3 pins of the switch facing forward, while aligning the grey cable to push into the PUF connection on the sensor board (Fig 6F).
- Solder the PUF connection (red circle in Fig 6G) and cut off any protruding wire.
- Push the switch down as far as possible (white arrow in Fig 6H) and solder the black cable to the centre pin as shown. Fix the switch to the 3D frame with small amounts of glue.
- Push the white cable through the hole on the left (Fig 6I). The assembly is finished on this side.

**Fig 6. Assembly of the light detector electronics v2 on the 3D printed frame.** (next page) (**A-I**) Sequential assembly of the components.


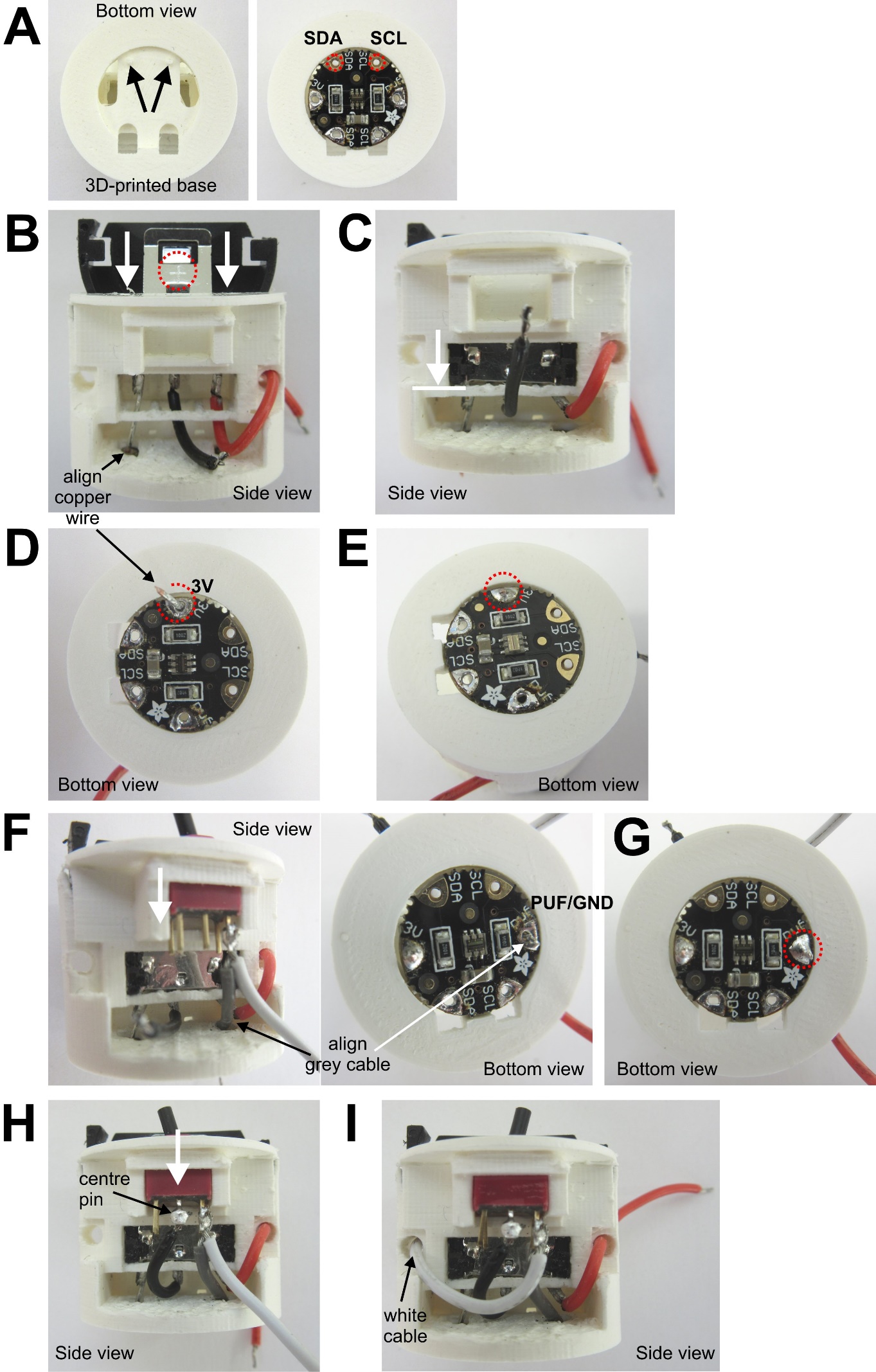


- Turn the unit around and put the RFduino unit into position as shown (Fig 7A).
- Align the green SCL and the yellow SDA cable with the connections on the sensor board (Fig 7B, C).
- Solder the SCL and SDA contacts as shown (red circles, Fig 7D).
- Solder the red and white cable to the RFduino (Fig 7E). If the RFduino is still able to move fix with a few small drops of superglue at the edges but away from any electrical contacts. This concludes the assembly of the sensor unit.
- Fig 7F shows different orientations of the finished unit.
- If a neutral density filter is required mount that in the tube lens first as demonstrated in Fig 4B.
- Slide in the sensor unit (Fig 7G).
- Screw in the retaining ring to keep the unit firmly in place (Fig 7H).
- Insert the CR2032 battery with the + side facing the switch as shown (Fig 7I).

The indentations on the top side of the 3D frame indicate the switch status: |=ON, ●=OFF.

- Test the assembly by switching on the unit. Run the IntensityCheck App to connect to the sensor or check whether a Bluetooth device called “LightSensor” is present.

**
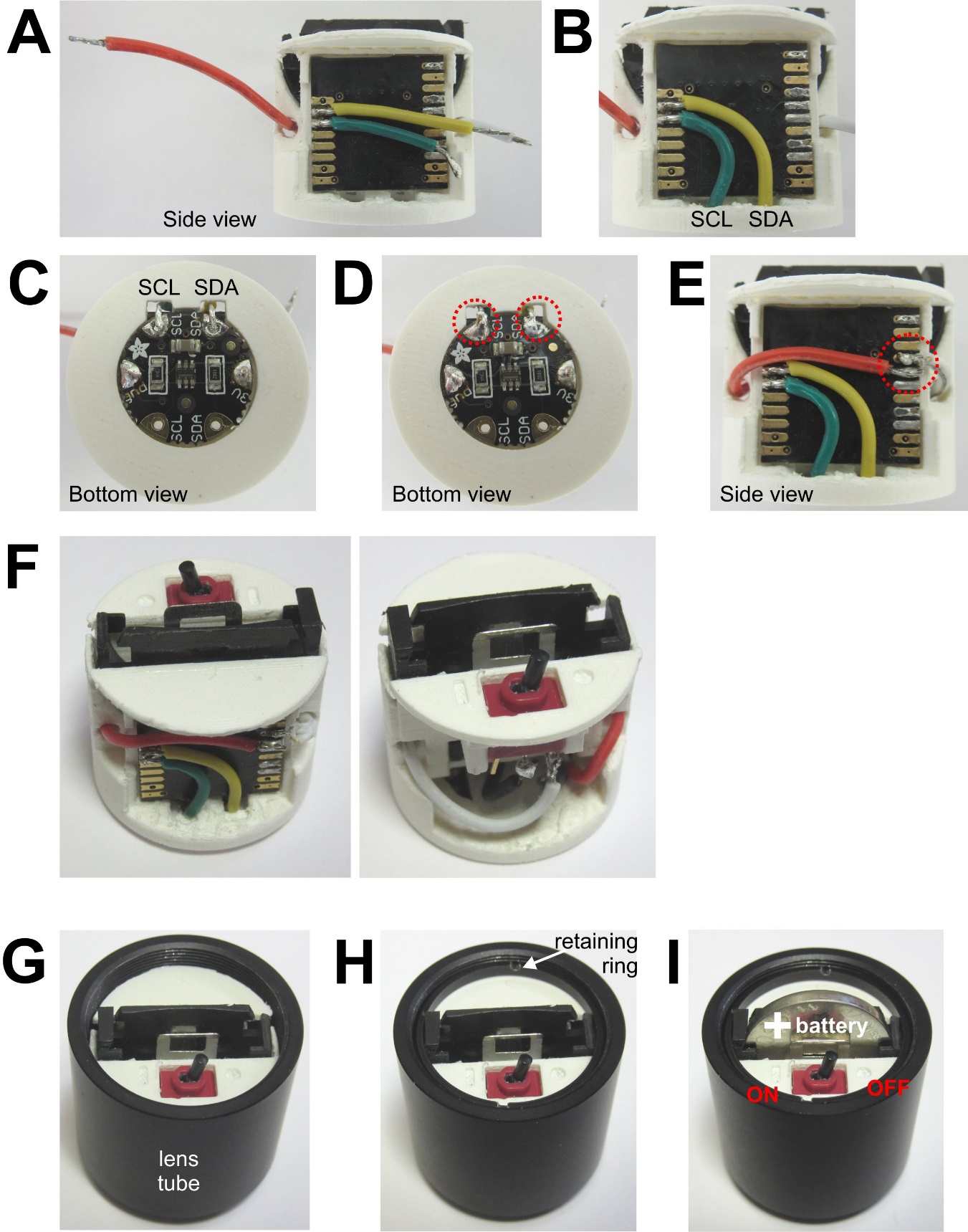
**

**Fig 7. Assembly and mounting of the light detector electronics v2.**

(**A-E**) Sequential assembly of the components. (**F**) Different views of the finished sensor unit. (**G**-**I**) Mounting the sensor in the lens tube.

# The IntensityCheck Android App

The IntensityCheck App has been developed for the Android operating system as those smartphones and tablets are cheap and readily available. The main hardware requirement is a Bluetooth low energy connection (BLE, Bluetooth Smart or Bluetooth 4.0 or higher) which is supported by Android versions 4.3 and higher. We have tested the software on a number of low cost devices (Motorola Moto E, Android version 5.0; Motorola Moto G 3^rd^ generation, Android version 6.0.1).

**Installation of the Android App**

- To install the App on an Android device you need to temporarily allow the installation of apps from unknown sources in the Security Settings of your device.


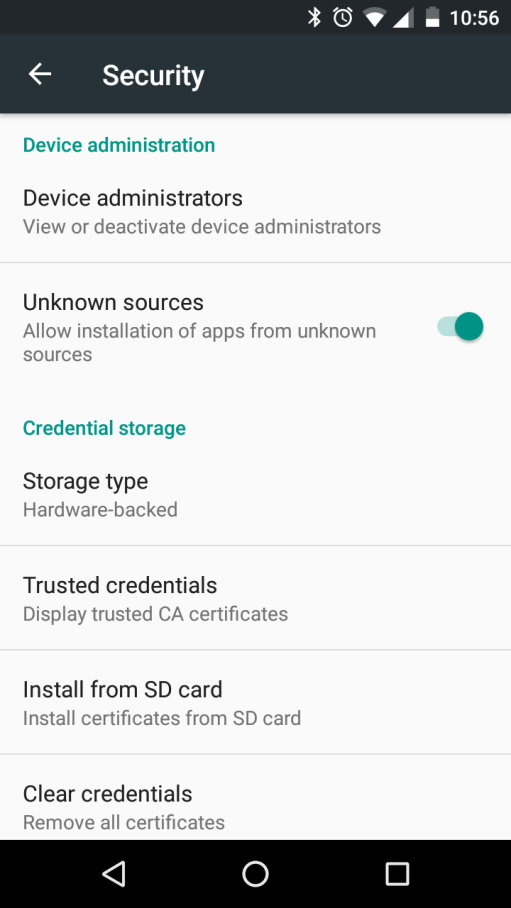


- The easiest way to install the App is to use a Gmail account to send the *IntensityCheck.apk* file as email attachment. On the Android device open the email and tap on the *IntensityCheck.apk* attachment.
- Once downloaded the system will open the Package Installer. Press either button and the App will be installed.


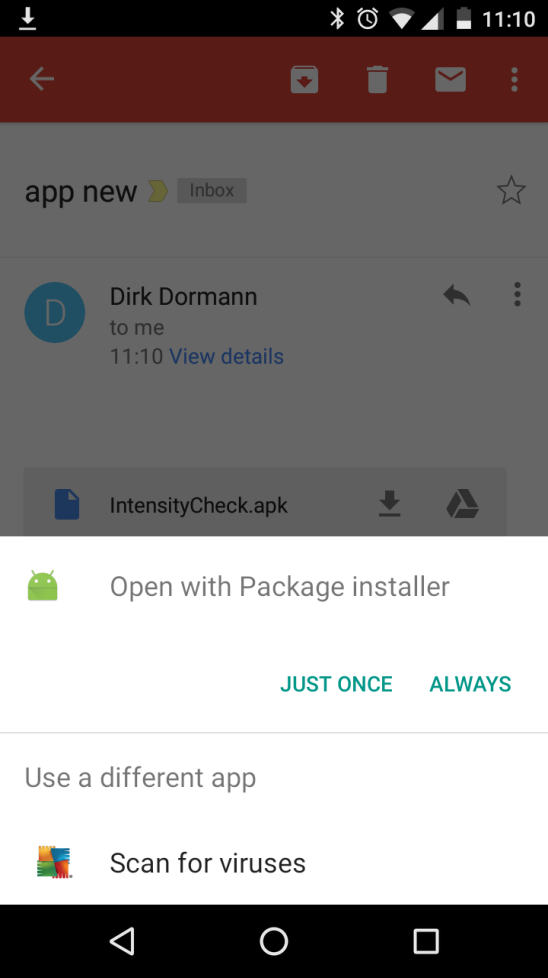


- The IntensityCheck App should appear on your screen:


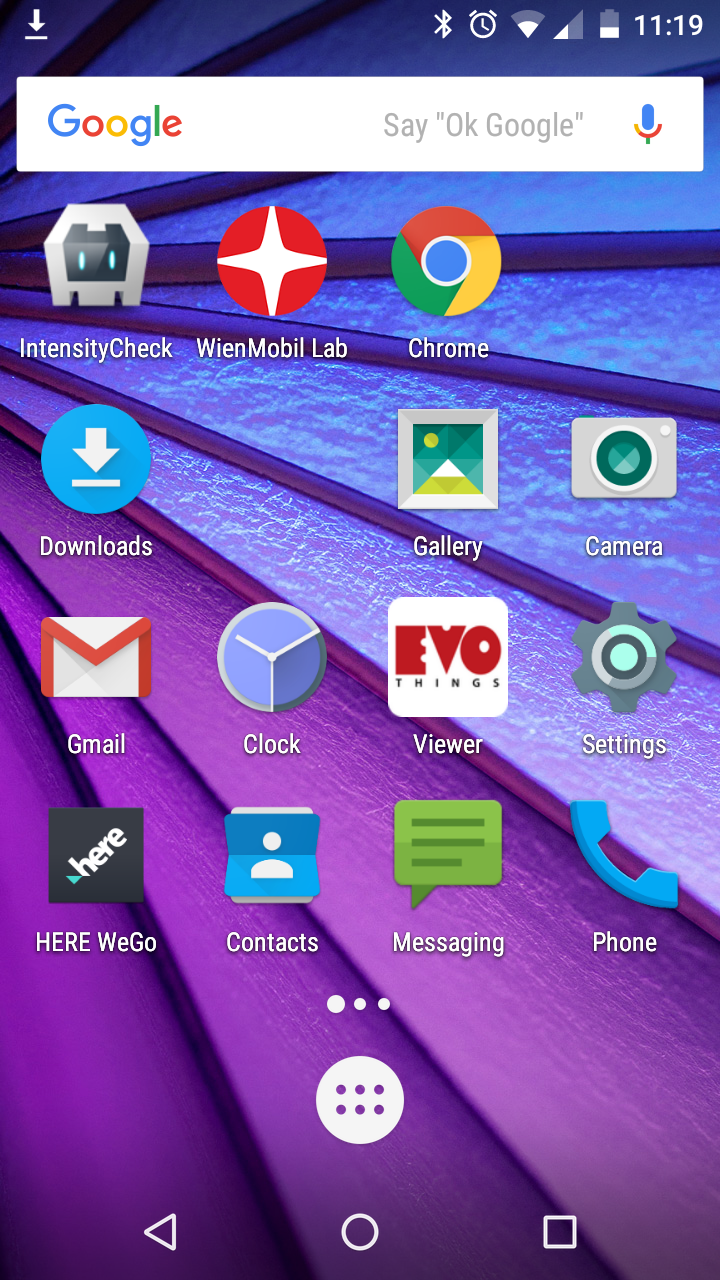


**Using the IntensityCheck App**

- Switch on the Bluetooth connection and Location as the App will not work without that. Bluetooth seems to require the location information.
- The smartphone display settings should be set to allow a font size up to “Large”, “Huge” can cause problems with the visibility of the buttons and the plotted graphs.
- Switch on the light sensor.
- Tap on the IntensityCheck App. Press the yellow CONNECT button to establish the connection with the sensor. If the connection fails, try again. If the connection cannot be made check whether the sensor is switched on or whether the battery needs to be replaced.


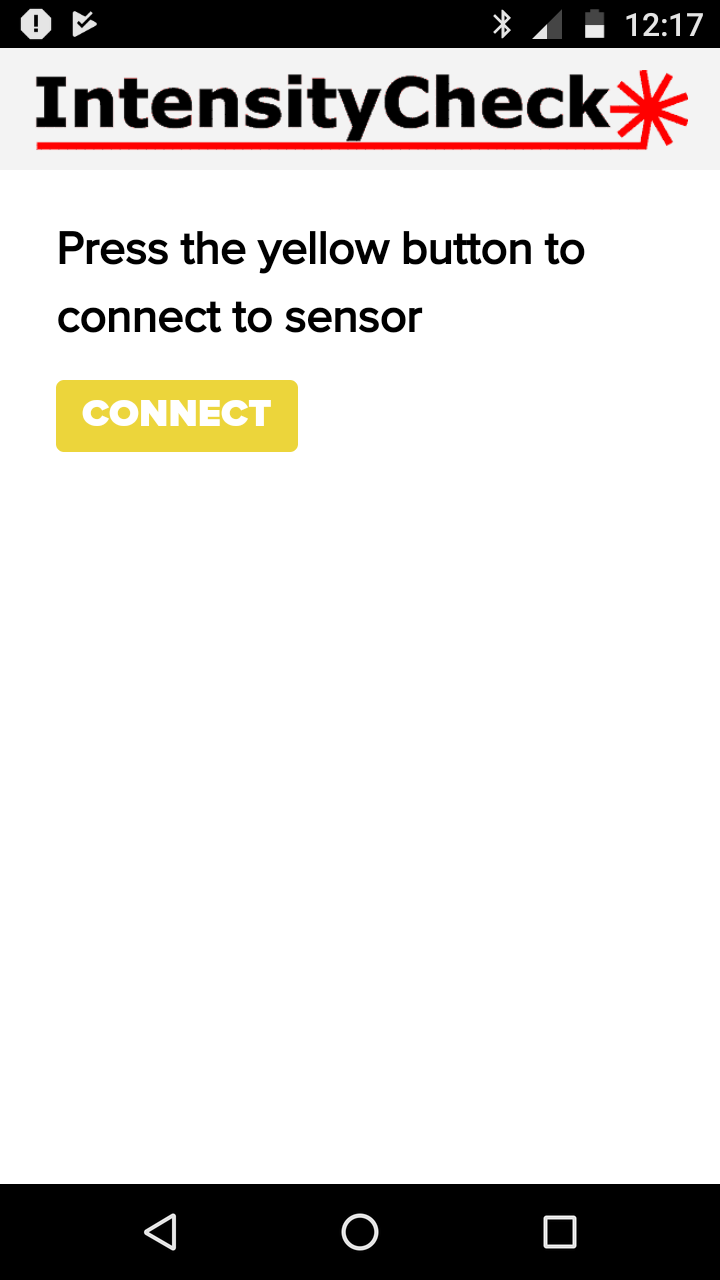


- A screen like the following should appear, here the display for the colour sensor is shown:

Toggle between FAST (low sensitivity) and SLOW (high sensitivity ) sensor readout speed

Mean Intensity

Close connection to sensor

**
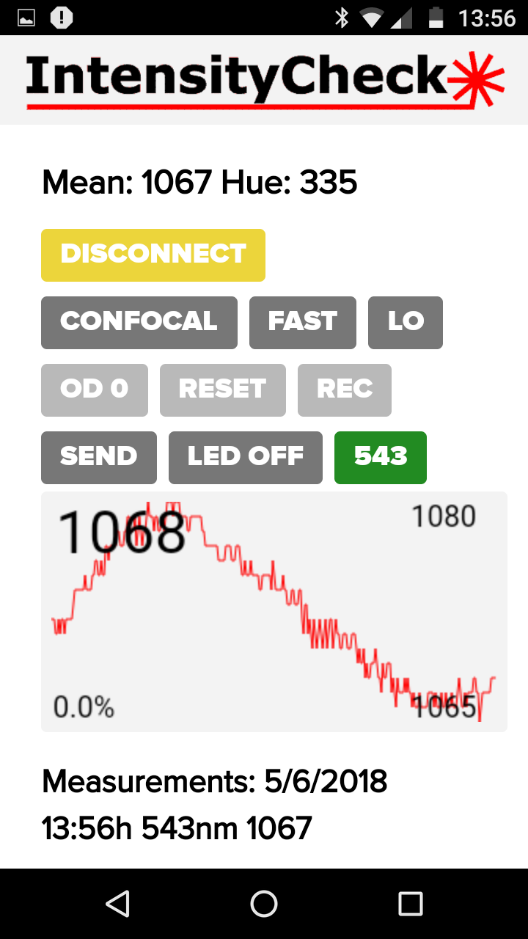
**

Autoscaled graph of recent intensity readings, update rate depending on sensor readout speed

% Difference between the last readings

Live Intensity measurement

Maximum and Minimum intensity readings

Send captured measurements by email and deletes all captured data

Resets intensity plots and deletes all captured data

When using colour sensor displays laser line or filterset. Tap to capture current intensity reading.

Toggle between live measurement display and timelapse recordings between HI (16x analog sensor gain) and LO (low, 1x gain)

Toggle between HI (16x analog sensor gain) and LO (low, 1x gain)

Tap to select the used neutral density filter (OD0-OD3), the measurements are adjusted accordingly

Toggle between CONFOCAL and WIDEFIELD Mode. Relevant when using colour sensor for display of laser wavelength or filtersets

Details of the captured measurements: date/time/wavelength/intensity

When using the colour sensor toggles the on-board LED between ON and OFF

- When the *REC* button is pressed once, the graph displays the time-lapse recordings that are automatically acquired every 5 seconds. The actual measurements will also be displayed below the graph. If the *REC* button is pressed again it will show the last 1000 measurements. The label *REC* will also change to *LIVE* as pressing it again will revert back to the live display showing the most recent 200 readings.


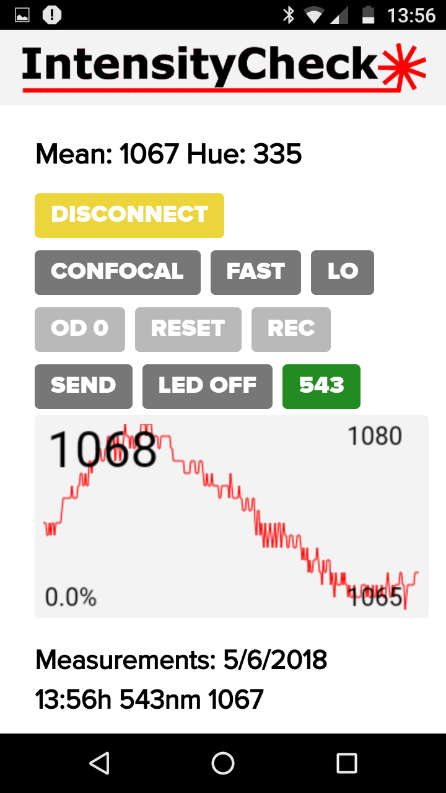

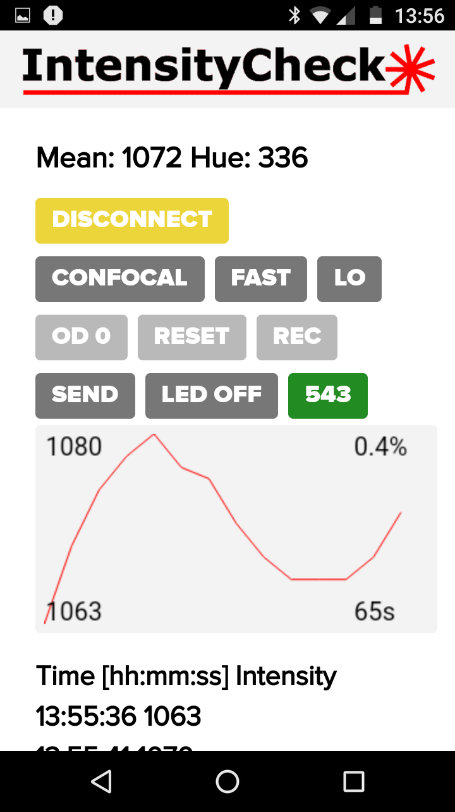

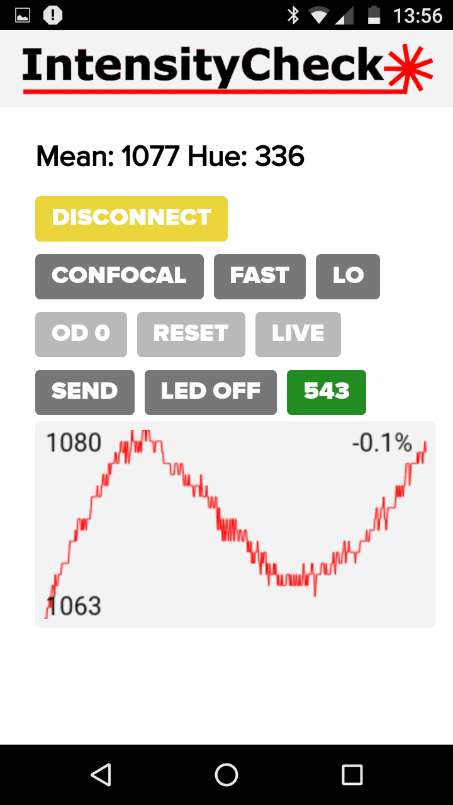


**Last 1000**

**Time lapse Graph**

**Live Graph**

- If you need to save any of the captured intensity readings or time lapse data press the *SEND* button on the corresponding screens to share the data by email. To capture the graphs simply take a screenshot. On an Android device this can usually be achieved by pressing and holding the power and volume-down buttons together for one to two seconds. Send the screenshot by email.

**Modifying and compiling the Android App**

The IntensityCheck App was developed using the free edition of Evothings Studio environment (<http://www.evothings.com/>, version 2.0.0 Beta3) which allowed the rapid prototyping and testing of the App without the need for frequent recompiling of the code. IntensityCheck is based on some Evothings code examples and templates that have been released under an open source Apache 2 license (<https://evothings.com/pricing/>).

Adobe Dreamweaver CS5.5 was used for code editing.

The final standalone version was compiled using the free and open source Apache Cordova (<https://cordova.apache.org/>), the installation and use of Cordova is described in detail on the Evothings website (<https://evothings.com/doc/build/cordova-guide.html>).

All the required source files are available for download (*IntensityCheck.zip*), the main source code is in *index.html*.

- Create a new Cordova project as described (<https://evothings.com/doc/build/cordova-guide.html>)
- Delete the contents of the www folder and copy the files from the IntensityCheck project (extract the files from *IntensityCheck.zip*)
- Add the relevant Cordova plugins to the project and the instructions for Android, this only has to be done once

In the Windows command line window change to the project folder and then issue the following commands, followed by pressing *ENTER*:

cordova plugin add cordova-plugin-rfduino [ENTER]

cordova plugin add cordova-plugin-ble-central [ENTER]

cordova plugin add cordova-plugin-ble [ENTER]

cordova plugin add cordova-plugin-bluetoothle [ENTER]

cordova platform add android [ENTER]

- To compile the android App issue the following command:

cordova build android [ENTER]

- The compiled App is called *android-debug.apk* and is found in the *...\platforms\android\build\outputs* folder.
- Transfer to the Android smartphone or tablet via Gmail and install as described above.
