# Supplementary material for: IntensityCheck – The light measuring app for microscope performance checks and consistent fluorescence imaging
Source: PLoS One. 2019 Mar 28;14(3):e0214659. doi: 10.1371/journal.pone.0214659 (PMC6438524; doi:10.1371/journal.pone.0214659)
Supplement: S3 File — (ZIP) [file pone.0214659.s010.zip › www/index.html]

IntensityCheck


Initializing...

 Connect 
 Disconnect 
  
 Confocal 
 FAST 
 LO 
  
 OD 0 
 Reset 
 Rec 
  
 Send 
 LED OFF 
 Wavelength 
  
